# Controlled intra- and extracellular localization of bioorthogonal polymeric nanozymes

**DOI:** 10.1039/d5sc07223a

**Published:** 2026-01-02

**Authors:** Cristina-Maria Hirschbiegel, Mathangi Shrikanth, Yagiz Anil Cicek, Nourina Nasim, Joe Truong, Junwhee Yang, Alexander Ribbe, Maged Abdelaziz, Vincent M. Rotello

**Affiliations:** a Department of Chemistry, University of Massachusetts Amherst 710N. Pleasant St. Amherst MA 01003 USA rotello@umass.edu; b Department of Polymer Science and Engineering, University of Massachusetts 120 Governors Drive Amherst MA 01003 USA

## Abstract

Bioorthogonal chemistry enables non-native chemical reactions to occur within complex biological environments. Transition metal catalysts mediate bioorthogonal uncaging reactions, allowing for the localized activation of chemically caged drug molecules. The ability to perform uncaging reactions selectively intra- or extracellularly expands the bioorthogonal toolkit by fine-tuning the localization of drug activation. However, transition metal complexes can diffuse through the cell membrane and, therefore, often lack control over intra- or extracellularly localized catalysis. Amphiphilic polymer nanoparticles can encapsulate transition metal catalysts, creating “polyzymes”. The polymer nanoscaffolds can be engineered to localize intra- or extracellularly by decorating the nanoparticle surface through surface charge. We designed polyzymes featuring either positive or negative surface charge, demonstrating cellular uptake and catalysis with cationic polyzymes and limited uptake and extracellular catalysis with negatively charged counterparts. Additionally, we performed the simultaneous catalytic activation of a quenched derivative of the anticancer drug Mitoxantrone to demonstrate the therapeutic potential of concurrent intra- and extracellular bioorthogonal catalysis. Our results indicate a significant improvement in cancer cell killing when combining intra- and extracellular drug activation.

## Abstract 2

Polymer-based bioorthogonal nanozymes selectively mediate catalysis intra- or extracellularly, allowing for control over the intra- or extracellular localization of catalysis.

## Graphical abstract

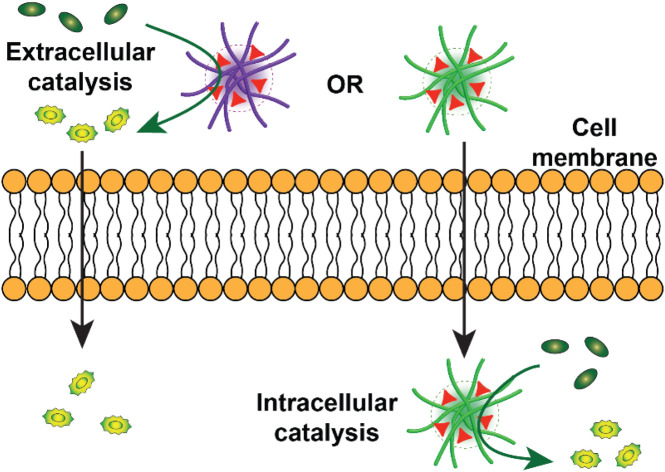

## Introduction

Bioorthogonal chemistry allows for non-native chemical reactions to occur within biological environments without disrupting native biological processes.^1–3^ Drug molecules can be chemically quenched through caging elements, yielding therapeutically inactive prodrugs.^4^ The localized uncaging of prodrugs within the target tissue increases the therapeutic efficacy of anticancer therapeutics and substantially decreases off-target effects of the drug.^5^ Bioorthogonal reactions can be facilitated using transition metal catalysts (TMCs), which can act as local “drug factories” by producing the drug inside the target tissue on demand.^6^ In particular, bioorthogonal uncaging reactions mediated by TMCs are promising due to the often straightforward synthesis of prodrugs based on FDA-approved drugs by introducing caging elements, such as allyl carbamate or propargyl moieties, and the high specificity of the respective uncaging reaction.^7,8^

Intra- and extracellular control over bioorthogonal catalysis is often critical for therapeutic and imaging applications.^9,10^ For instance, bioorthogonal catalysts can activate therapeutics that possess intracellular targets, such as the nucleus and mitochondria, or extracellular targets, such as cell surface signaling pathways or necrotic tumor areas.^11–13^ Additionally, prodrugs may be cell membrane-permeable or membrane-impermeable, requiring the appropriate intra- or extracellular localization of the TMC for bioorthogonal activation.^14^ However, free TMCs are small molecules that can often diffuse through tissue and cell membranes.^15^ Therefore, free TMCs generally lack control over intra- or extracellularly localized catalytic activity.^16^

TMCs can be encapsulated into nanomaterials, such as inorganic and organic nanoparticles and metal–organic frameworks, creating bioorthogonal “nanozymes”.^17,18^ The nanomaterial scaffold enhances the solubility and catalytic performance of the catalyst in a biological environment and protects the catalyst from enzymatic degradation.^19^ Furthermore, the nanoscaffold provides an additional layer of modularity by controlling physicochemical features and incorporating targeting or responsive features.^20–22^

Amphiphilic polymer nanoparticles are highly biocompatible and possess low toxicity towards mammalian cells.^23,24^ TMCs can be encapsulated into polymeric nanoscaffolds, creating bioorthogonal “polyzymes” (PZs).^25^ The surface of these polymer nanoscaffolds offers design space for chemical moieties that promote the localized accumulation in disease areas or cellular compartments, for instance, by attaching targeting elements or introducing charged headgroups.^26,27^ For instance, positively charged polymer nanoparticles interact with the negatively charged cell surface glycans of mammalian cells, thereby promoting intracellular uptake, whereas negatively charged polymer nanoparticles are less uptaken due to charge–charge repulsion.^28^ In solid tumors, targeting the tumor microenvironment (TME) is an attractive strategy to enhance cancer killing, as the activated prodrug can diffuse through the cell membrane of cancer cells and kill them.^29^ In fact, negative surface charges of nanocarriers contribute to enhanced blood circulation time *in vivo* and slower renal clearance of the nanoparticle, which can enhance accumulation of the cargo inside the TME.^30^ Positive surface charges of nanocarriers promote intracellular uptake; however, nanoparticles can be trapped inside the endosome and experience enzymatic degradation in the late endosome/early lysosome.^31^ Additionally, intracellular thiols may quench the bioorthogonal catalyst over time, decreasing therapeutic efficacy and inhibiting prodrug activation.^32^ Therefore, spatial control of bioorthogonal catalysis is desired for the effective control over localized prodrug activation, and the combination of intra- and extracellular prodrug activation may enhance the therapeutic outcome.

**Fig. 1:**
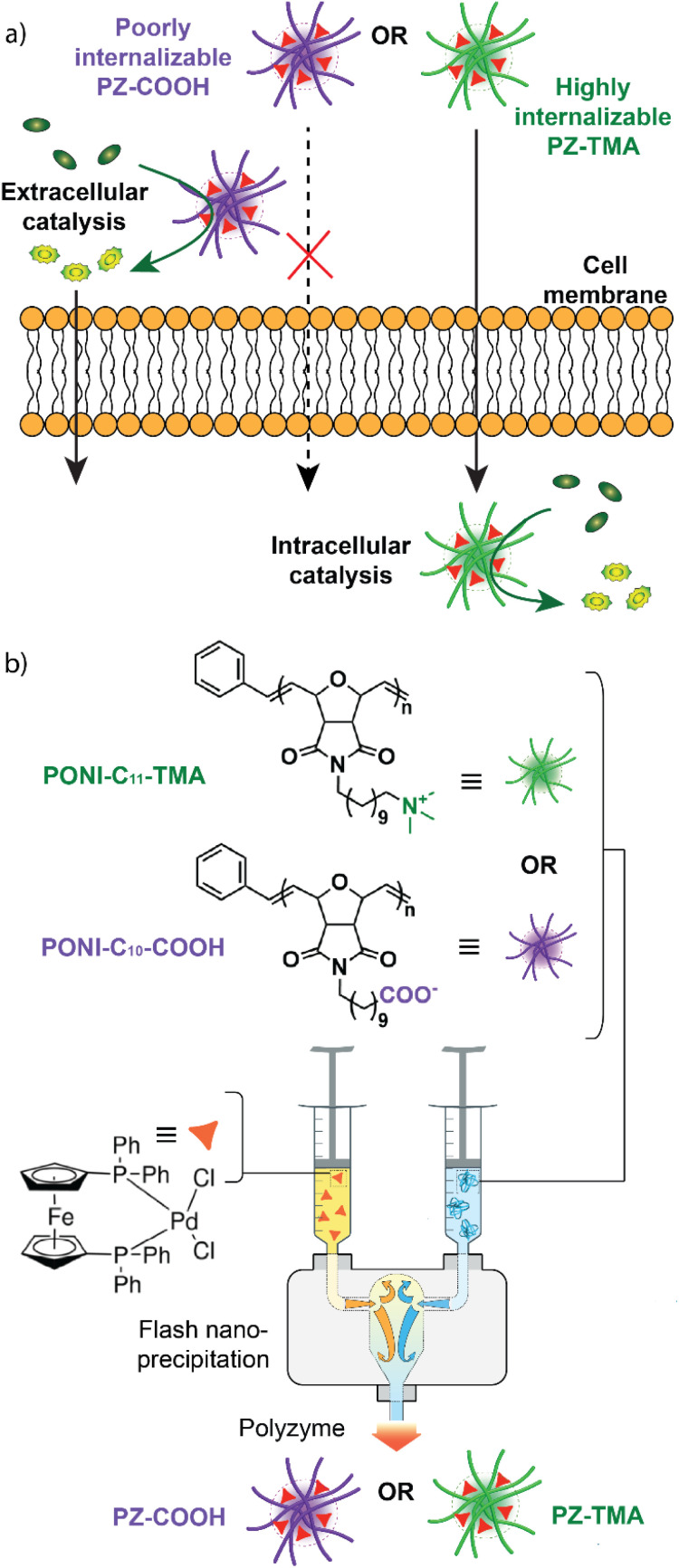
(a) Intra- or extracellular catalysis mediated by the positively (PZ-TMA) or negatively (PZ-COOH) charged PZ, respectively; (b) PZ preparation using flash nanoprecipitation (FNP) with either PONI-C_11_-TMA or PONI-C_10_-COOH and Pd(dppf)Cl_2_.

We designed and characterized two charged polyoxanorbornene imide-based polymer nanoscaffolds (cationic PONI-C_11_-TMA and anionic PONI-C_10_-COOH) and encapsulated a Pd-based TMC (Pd(dppf)Cl_2_) to generate two corresponding bioorthogonal PZs (cationic PZ-TMA and anionic PZ-COOH) (Fig. 1b). The respective PZs catalyze the bioorthogonal allyl carbamate-uncaging reaction of a quenched rhodamine 110 derivative either intra- (cationic) or extracellularly (anionic), allowing for spatial control of catalysis and enhanced pro-fluorophore activation when combined (Fig. 1a).

The catalytic performance towards the substrate was characterized by Michaelis–Menten kinetics, indicating higher catalytic conversion mediated by the positively charged PZ-TMA in the absence of serum proteins. However, a decrease in catalytic activity of PZ-TMA was observed in the presence of serum proteins, presumably due to the formation of a protein corona around the particle surface, decreasing substrate access.^33^ In contrast, the catalytic activity of PZ-COOH was decreased minimally, indicating retention of catalytic activity in serum-containing conditions. Intracellular pro-fluorophore activation was performed in HeLa cells and was concomitant with PZ uptake, measured by inductively coupled plasma mass spectrometry (ICP-MS). Additionally, when intra- and extracellular pro-fluorophore activation was combined, using PZ-TMA and PZ-COOH sequentially, a significant increase of intracellular fluorescence was observed, indicating improved fluorophore accumulation within cells.

We demonstrated the therapeutic potential of this combined approach by catalytically uncaging a derivative of the clinically used anticancer drug mitoxantrone (pro-Mitox), which targets the intracellular enzyme topoisomerase II.^34^ The negatively charged PZ performed cell killing more efficiently than the positively charged PZ despite the intracellular activation of the prodrug mediated by PZ-TMA, presumably due to substantially more prodrug activation mediated by PZ-COOH compared to PZ-TMA. Significantly, combined intra- and extracellular catalysis showed more efficient cancer-killing compared to the respective treatment with either the intra- or extracellular approach, which can be explained by higher product concentrations due to the simultaneous intra- and extracellular prodrug activation. These results demonstrate that a combined intra- and extracellular strategy could offer a promising path for more efficient bioorthogonal anticancer treatment.

## Results and discussion

### Polyzyme fabrication and characterization

Bioorthogonal PZs were fabricated using either cationic (PONI-C_11_-TMA) or anionic (PONI-C_10_-COOH) polymer nanoscaffolds, consisting of a polyoxanorbornene imide backbone and a hydrophobic carbon chain that form the interior hydrophobic core of the nanoparticle, and a charged headgroup (either carboxylate or trimethyl amine) that provides water solubility and interacts with the biological environment.^27^ The charged polymers were synthesized using ring-opening metathesis polymerization (ROMP),^35^ yielding polymer nanoscaffolds of similar molecular weight (32 kDa for PONI-C_11_-TMA and 38 kDa for PONI-C_10_-COOH) (Fig. S3–S16). The selected Pd catalyst (Pd(dppf)Cl_2_) is insoluble in water and can be encapsulated into the hydrophobic interior core of the respective nanoparticle.^36^

The charged PZs were fabricated using flash nanoprecipitation (FNP, Fig. 1b).^37^ Briefly, the respective polymer was dissolved in MilliQ water at a concentration of 1 mg mL^−1^, and 0.6 mL of the solution was loaded into a 1 mL syringe. A second syringe was loaded with 0.6 mL of a solution of Pd(dppf)Cl_2_ dissolved in a 1 : 1 mixture of acetone and dimethyl sulfoxide. Both syringes were attached to the inlets of a confined impinging jet (CIJ) mixer and rapidly simultaneously impinged. The resulting charged polyzymes PZ-TMA and PZ-COOH were collected in a 6 mL quench bath consisting of either MilliQ water (for PZ-TMA) or a 50 µM solution of NaBH_4_ in MilliQ water (for PZ-COOH) to stabilize the negative charge, and the organic solvent was removed by centrifugation using a centrifuge filter (30 kDa).

**Fig. 2:**
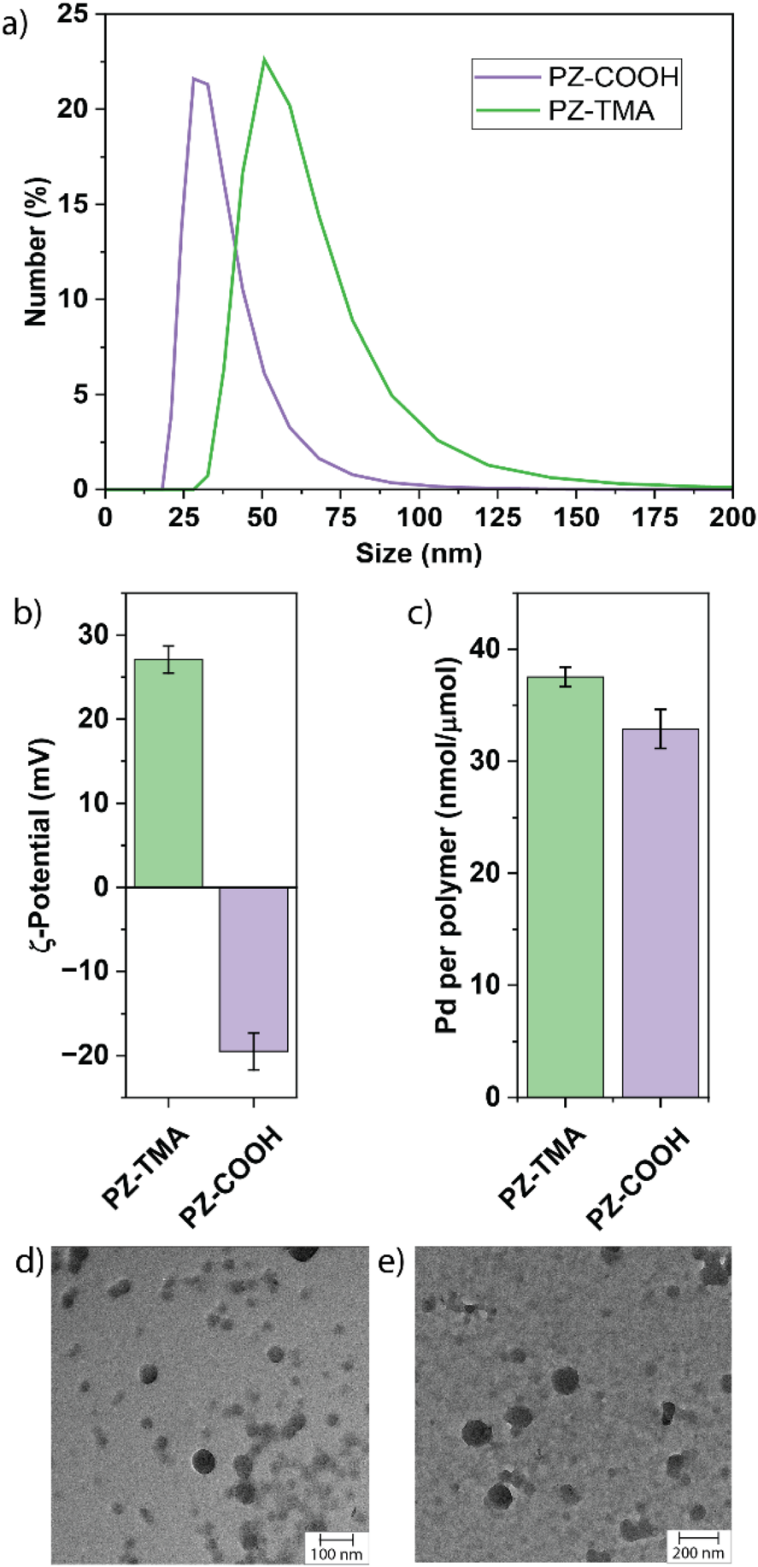
(a) Size distribution measured by dynamic light scattering (DLS); (b) *ζ*-potential of respective PZs; (c) quantification of encapsulated Pd^106^ measured by inductively coupled plasma mass spectrometry (ICP); TEM images of (d) PZ-TMA and (e) PZ-COOH.

The size of the resulting PZs was determined by dynamic light scattering (DLS, Fig. 2a). Notably, PZ-TMA was larger than PZ-COOH, indicating differences in the folding pattern between the respective polymers during encapsulation.^38^ Additionally, transmission electron microscopy (TEM) was employed to observe the shape and size of the nanoparticles, showing spherically shaped particles and Pd encapsulation in the high-contrast areas (Fig. 2d and e). Energy-dispersive X-ray spectroscopy (EDS) was used to visualize the distribution of Pd within the nanoparticle (Fig. S17 and S18). The results indicate a homogeneous distribution of Pd and the ferrocenyl-containing ligand across PZ-TMA and PZ-COOH, indicating that the encapsulated catalyst remains assembled after PZ fabrication.

The surface charge of the respective PZs was determined by measuring their *ζ*-potential (Fig. 2b). As expected, the respective surface charges of the two PZs were either positive for PZ-TMA or negative for PZ-COOH, indicating appropriate folding of the nanoscaffold. Finally, the catalyst loading was determined by ICP-MS, showing similar encapsulation of Pd per polymer (Fig. 2c and S19). Additionally, the polyzyme stability and catalyst retention were determined using ICP-MS after filtration of the respective polyzyme 5 days after fabrication (Fig. S20) to remove any precipitated catalyst. Excellent catalyst retention was observed for PZ-TMA (98%), while minimal loss of catalyst (83%) was observed for PZ-COOH, presumably due to differences in polyzyme folding and solubility, leading to loss of catalyst or polyzyme over time.

### Kinetic performance of polyzymes in solution

**Fig. 3:**
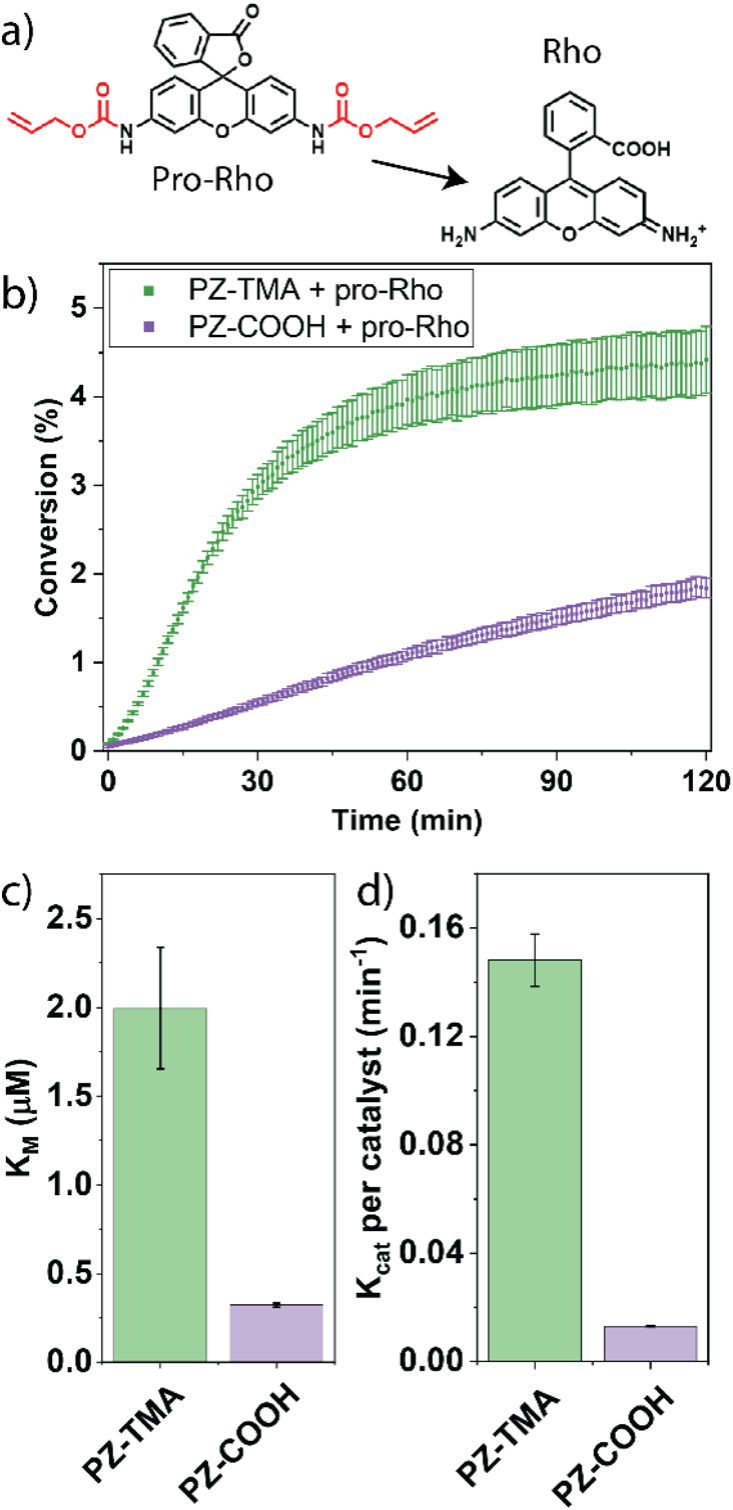
(a) Allyl carbamate uncaging reaction mediated by PZs; (b) kinetic performance of PZ-TMA and PZ-COOH with pro-Rho; (c) Michaelis–Menten constant *K*_M_; (d) catalytic constant *K*_cat_ per catalyst.

The kinetic performance of the respective PZs was determined by catalytically uncaging an allyl carbamate-caged derivative of rhodamine 110 in phosphate–buffered saline (PBS), thereby reestablishing fluorescence (Fig. 3a).^39^ Interestingly, the PZ-TMA plateaued substantially faster than PZ-COOH, achieving a higher catalytic conversion, presumably due to easier accessibility of the catalyst and substrate diffusion within the polymer scaffold, as indicated by a larger particle size (Fig. 3b).^40,41^ Additionally, the catalytic performance was characterized *via* Michaelis–Menten kinetics by maintaining a constant PZ concentration, varying the substrate concentration, and calculating the resulting Michaelis–Menten constant (*K*_M_) and maximum velocity (Fig. 3c and S21). Compared to PZ-COOH, PZ-TMA demonstrated a higher *K*_M_ value, indicating a lower binding affinity of the substrate to the PZ and catalyst saturation at higher substrate concentration.^42^ These results indicate that PZ-TMA exhibits greater catalyst accessibility to the substrate compared to PZ-COOH. Additionally, we calculated the catalytic turnover (*K*_cat_) of each respective PZ per catalyst and found that the Pd catalyst within PZ-TMA performs a significantly larger number of catalytic turnovers, which may be attributed to improved catalyst access within the nanoscaffold (Fig. 3d) due to differences in the folding pattern of the respective PZs.

**Fig. 4:**
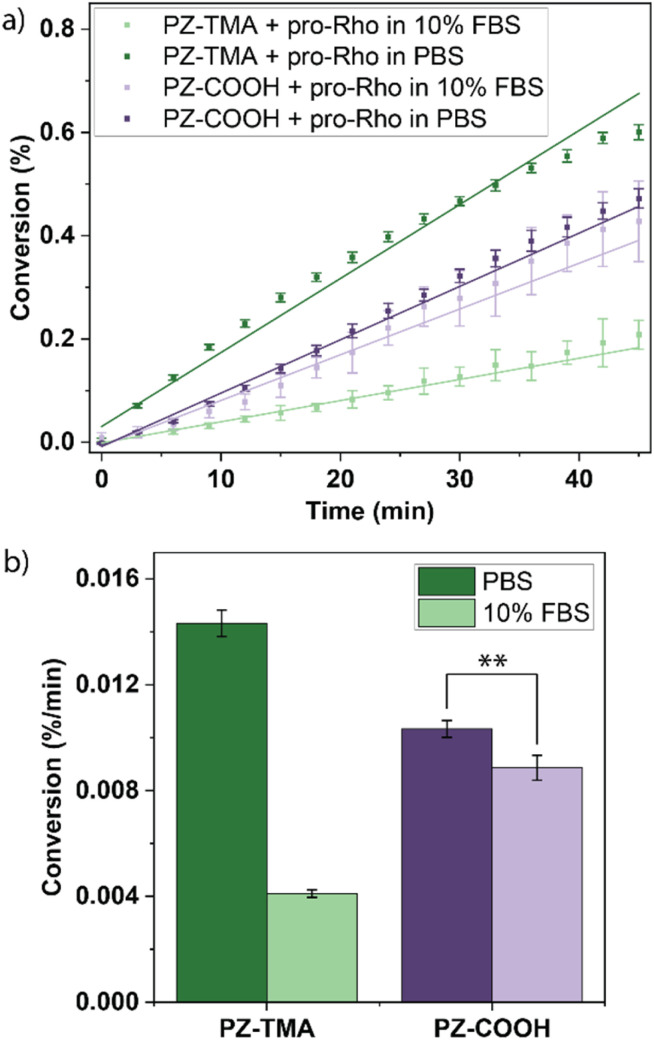
(a) Catalytic activation of pro-Rho in PBS or PBS containing 10% fetal bovine serum (FBS) mediated by PZ-TMA or PZ-COOH; (b) reaction rate (slope of linear fit) of the respective uncaging reactions in (a) demonstrating the impact of protein corona formation on PZ activity. All values represent the average of six individual measurements; error bars represent the standard deviation. Statistical analysis was performed using Student's *t*-test. ** = *p* < 0.01.

Thereafter, the catalytic performance of each PZ was compared in the presence and absence of 10% fetal bovine serum (FBS) by monitoring the catalytic uncaging of pro-Rho (Fig. 4a). While PZ-TMA performs catalysis more efficiently than PZ-COOH in the absence of FBS, the presence of serum proteins significantly impacts the catalytic performance of PZ-TMA. Meanwhile, the catalytic performance of the negatively charged PZ-COOH is impacted to a substantially lesser extent than that of PZ-TMA (Fig. 4b). This observation can be explained by the formation of a stronger protein corona of negatively charged serum proteins around PZ-TMA compared to PZ-COOH.^43^ These findings suggest that the use of negatively charged PZs can improve catalysis in a serum-containing environment compared to positively charged PZs by mitigating the effects of protein corona formation on catalytic performance. Additionally, we tested the catalytic performance of the respective nanozymes in the presence of common intracellular nucleophiles, such as glutathione and cysteine (Fig. S22), to further mimic the impact of the cellular environment on catalytic performance.^44,45^ As expected, the catalytic performance of both nanozymes improved in the presence of these nucleophiles due to the acceleration of the Pd-mediated Tsuji–Trost reaction.^46,47^ Furthermore, the presence of glutathione enhanced the catalytic performance of PZ-TMA more than cysteine (Fig. S22c), presumably due to the negative charge of the molecule at physiological pH and therefore enhanced access to the catalyst embedded within the nanoscaffold.^48,49^ Meanwhile, the presence of cysteine enhanced the catalytic performance of PZ-COOH more than glutathione (Fig. S22d), presumably due to the overall neutral charge of cysteine at physiological pH and charge–charge repulsion between the polymer nanoscaffold and glutathione.^48,49^ However, it is important to note that while catalysis is initially accelerated, the presence of intracellular thiols could lead to catalyst deactivation due to catalyst poisoning over time, quenching the catalytic activity of the nanozyme.^50^

### Kinetic performance of polyzymes inside and outside of cells

**Fig. 5:**
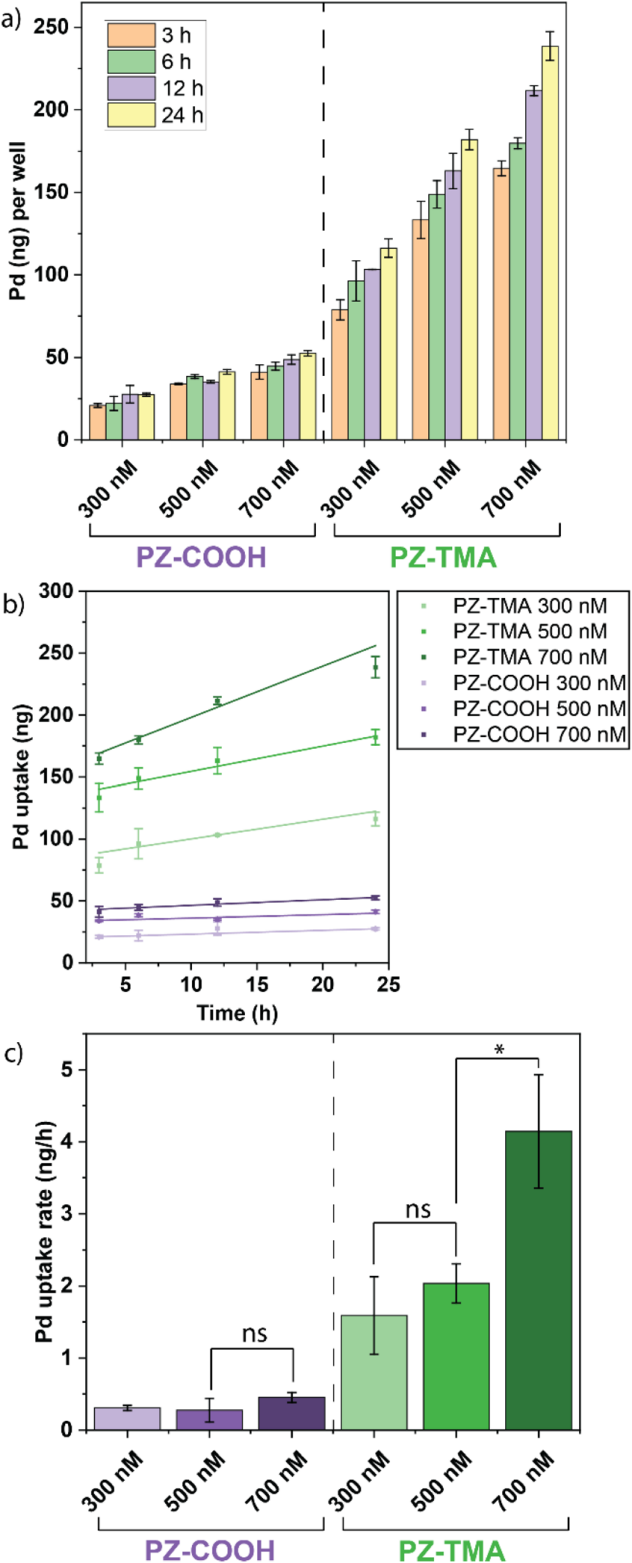
(a) Quantification of Pd^106^ in HeLa cells by ICP-MS at different time points of incubation with respective PZ; (b) linear fit of time-dependent uptake within cells; (c) average uptake of Pd (ng h^−1^) in HeLa cells per PZ at different concentrations, demonstrating significantly higher uptake rates of positively charged PZ-TMA. All values represent the average of three individual measurements; error bars represent the standard deviation. Statistical analysis was performed using Student's *t*-test. ns = not significant; * = *p* < 0.05.

After characterizing the physical properties and catalytic performance of the PZs in solution, we determined the catalytic behavior *in vitro* on HeLa cells. In particular, we studied the differences in intracellular uptake and catalysis based on the surface charges of the PZs by quantifying the respective uptake using ICP-MS and performing the intracellular activation of pro-Rho. All experiments were performed in serum-containing cell culture medium. Briefly, HeLa cells were seeded on a 24-well plate 24 h prior to treatment with PZs at different concentrations (300/500/700 nM) for different durations to quantify the intracellular uptake of the respective PZs over time by ICP-MS (Fig. 5a and b). As expected, the positively charged PZ-TMA was significantly more uptaken by HeLa cells than the negatively charged PZ-COOH at all concentrations due to interactions with the negatively charged glycocalyx on the cell surface, followed by intracellular uptake.^51,52^ Furthermore, the uptake of Pd per hour (across the duration of incubation) was averaged, indicating a similar uptake rate of PZ-TMA at 300 and 500 nM, but a significantly higher uptake rate at 700 nM (Fig. 5c). The difference in uptake rate can be explained by concentration-dependent uptake patterns.^53^ However, this increase in uptake rate was not observed for PZ-COOH. These results indicate that the positive surface charge of PZ-TMA facilitates interaction with the negatively charged cell membrane, resulting in intracellular uptake, while the negatively charged PZ-COOH is substantially less uptaken. The limited uptake of PZ-COOH can be explained by non-specific uptake over time.^54–56^

**Fig. 6:**
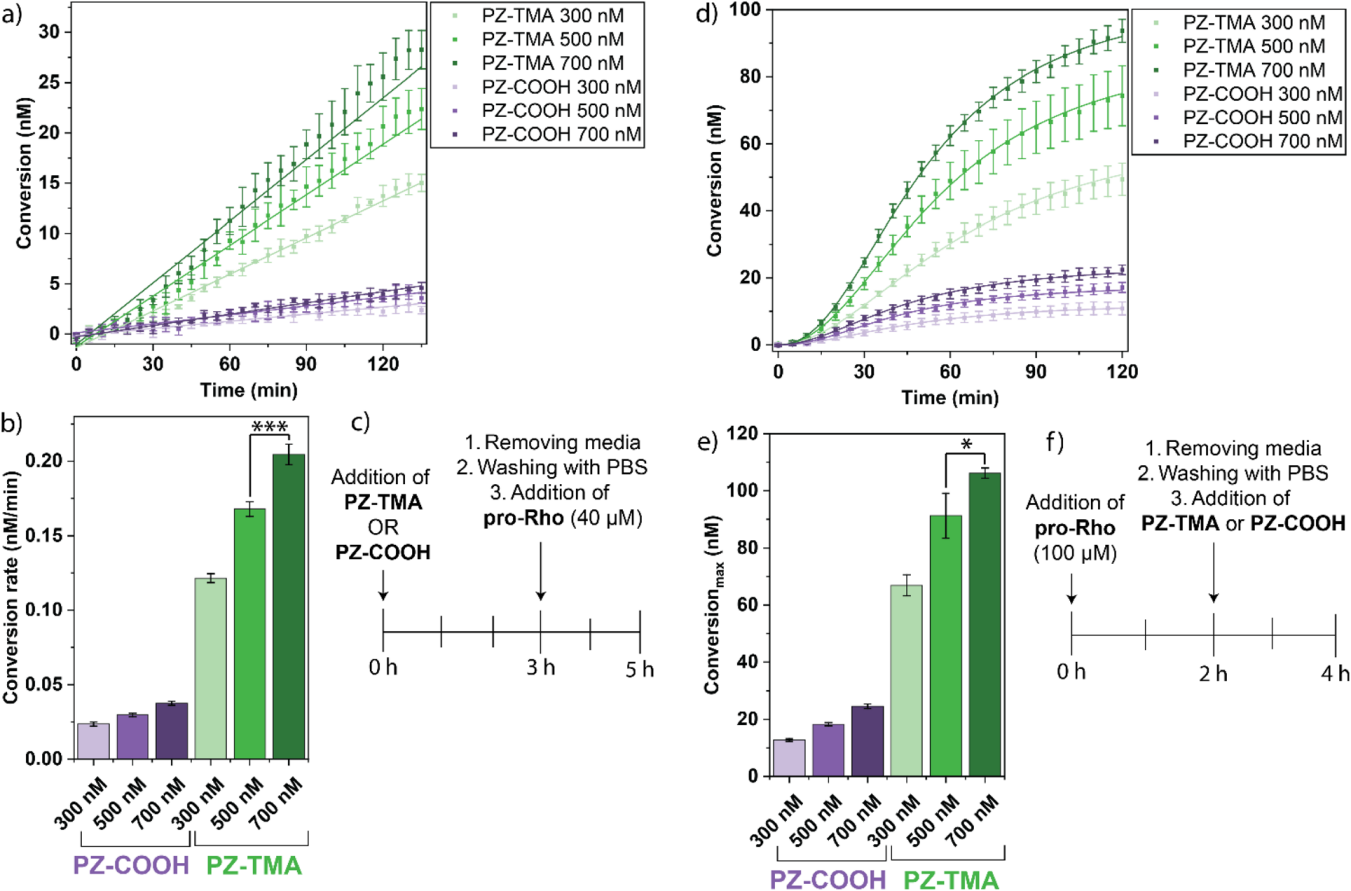
(a) Intracellular activation of pro-Rho after 3 h of incubation of HeLa cells with respective PZ and subsequent addition of pro-Rho (40 µM); (b) reaction rate (slope of linear fit) of the respective PZ of (a); (c) treatment timeline of (a) and (b); (d) catalytic activation of pro-Rho after 2 h of incubation of HeLa cells with pro-Rho (100 µM) and subsequent addition of respective PZs; (e) conversion maximum of the respective PZs at different PZ concentrations of (b) plotted using the Hill equation; (f) treatment timeline of (d) and (f). All values represent the average of four individual measurements; error bars represent the standard deviation. Statistical analysis was performed using Student's *t*-test. * = *p* < 0.05; *** = *p* < 0.005.

After confirming the charge-dependent intracellular uptake of the respective PZs, we evaluated the intracellular catalytic performance by incubating HeLa cells with either PZ-TMA or PZ-COOH at varying concentrations (300/500/700 nM) for 3 h, followed by washing the cells, adding pro-Rho, and monitoring the fluorescence (Fig. 6a and c). Alternatively, we performed a similar experiment by reversing the order of addition, first incubating HeLa cells with pro-Rho for 3 h, followed by washing and addition of the respective PZs at varying concentrations (300/500/700 nM) (Fig. 6b and d). Importantly, in both experiments, the intracellular activity of the respective polyzymes was studied by sequentially adding either (1) first PZ-TMA or PZ-COOH and second the pro-dye (Fig. 6a–c), or (2) first the pro-dye and second the respective polyzyme (Fig. 6d–f), to demonstrate the catalytic performance after the nanozymes were uptaken by the cells. Since the pro-dye was washed away before the addition of the respective polyzymes in case (2), higher concentrations of pro-Rho (100 µM) were selected to ensure appropriate uptake of the pro-dye. In both experiments, PZ-TMA demonstrated substantially more pro-Rho activation in a concentration-dependent manner, indicating uptake-dependent intracellular catalysis. Interestingly, when HeLa cells were first incubated with the respective PZs and pro-Rho was added afterwards, the reaction kinetics followed a linear increase in fluorescence. Meanwhile, when HeLa cells were first incubated with pro-Rho, and the respective PZs were added afterwards, the reaction kinetics followed a sigmoidal curve. This observation can be explained by the slow diffusion of the PZ into the cell compared to the rapid diffusion of the small molecule pro-Rho, presumably leading to a faster onset of catalysis when cells are first incubated with pro-Rho.

**Fig. 7:**
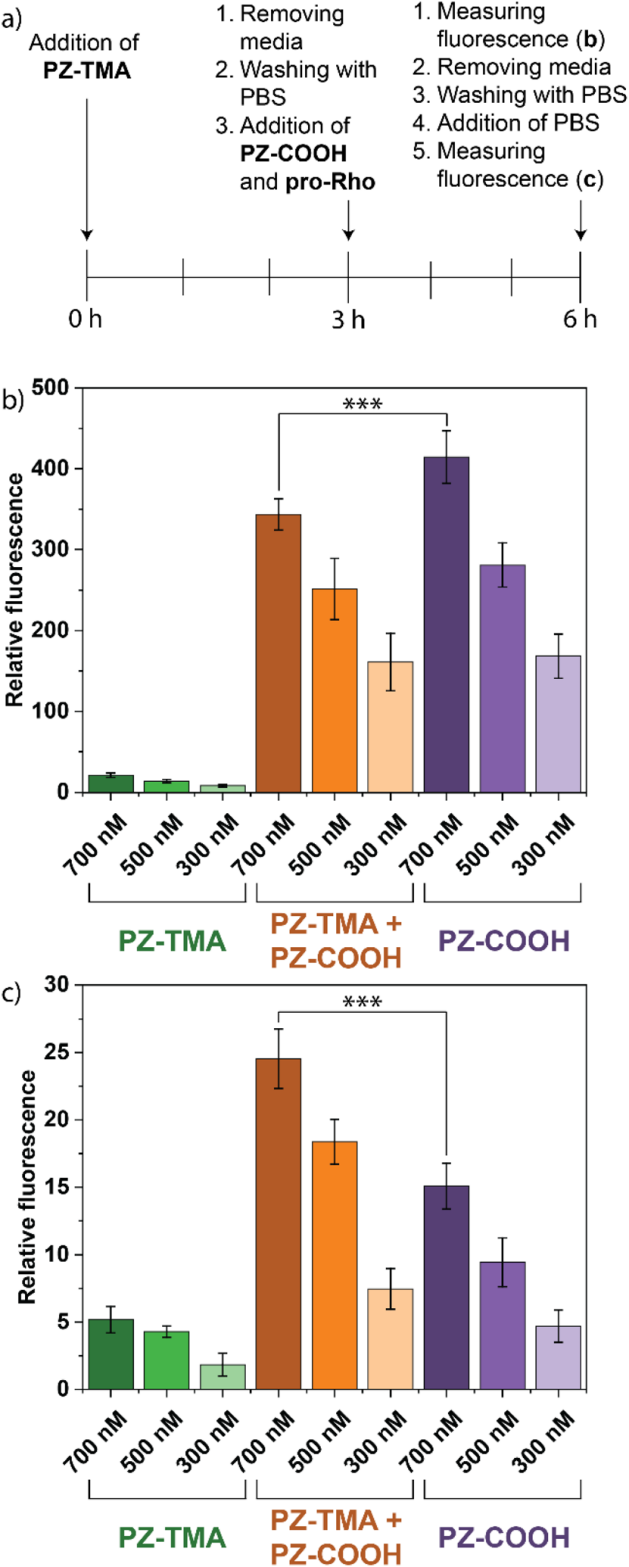
(a) Treatment timeline of (b) and (c); (b) intra- and extracellular fluorescence of HeLa cells incubated with PZ-TMA for 3 h, followed by the addition of pro-Rho and PZ-COOH and incubation for an additional 3 h; (b) intracellular fluorescence in HeLa cells after removing the media, washing, and adding fresh PBS. All values represent the average of six individual measurements; error bars represent the standard deviation. Statistical analysis was performed using Student's *t*-test. *** = *p* < 0.005.

After confirming intracellular uptake of PZ-TMA, we hypothesized that the combined catalytic activity of PZ-TMA intracellularly and PZ-COOH extracellularly could yield a significantly higher amount of intracellular product due to (1) the intracellular activation of substrate mediated by PZ-TMA and (2) the extracellular activation of substrate mediated by PZ-COOH, followed by substrate diffusion into the cell. We tested our hypothesis using HeLa cells by first incubating the cells with PZ-TMA for 3 h, followed by the addition of pro-Rho and PZ-COOH together, and measuring the fluorescence in the media (extracellular + intracellular) after 3 h of reaction time. Thereafter, the media was removed and replaced with fresh PBS, and the fluorescence was measured again to obtain only the intracellularly remaining fluorescence (Fig. 7a). As expected, cells incubated with pro-Rho and only PZ-COOH demonstrated significantly more fluorescence in the media than cells incubated with pro-Rho and PZ-TMA only, due to the rapid catalysis in the extracellular environment (Fig. 7b). Furthermore, after removing the media and measuring intracellular fluorescence, significantly higher Rho fluorescence was observed in cells treated with PZ-TMA + PZ-COOH compared to cells treated with the respective PZ alone (Fig. 7c), indicating that combined intra- and extracellular catalysis can increase the fluorophore concentrations within the cells.

### Intra- and extracellular combination therapy

**Fig. 8:**
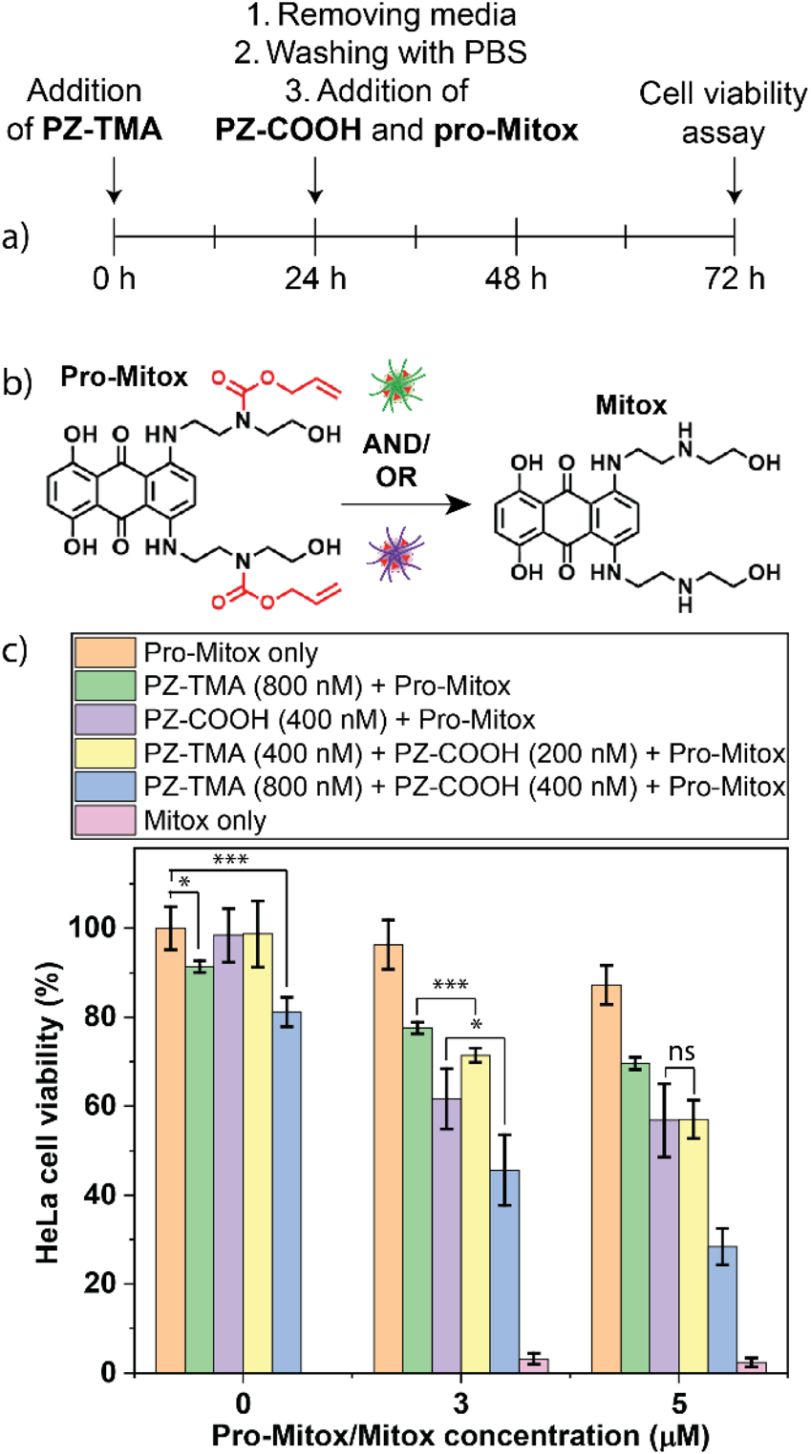
(a) Experimental timeline for cell killing studies; (b) bioorthogonal uncaging reaction of pro-Mitox in the presence of either PZ-TMA, PZ-COOH, or both simultaneously; (c) HeLa cell viability after treatment with pro-Mitox and either the respective PZs alone or both PZs together (50% or 100% concentration) demonstrates similar cell killing to PZ-COOH alone when treated with half of the concentration of each respective PZ, but enhanced cell killing when treated with the full concentration of each PZ simultaneously. All values represent the average of four individual measurements; error bars represent the standard deviation. Statistical analysis was performed using Student's *t*-test. ns = not significant; * = *p* < 0.05; *** = *p* < 0.005.

After observing higher intracellular fluorescence with a combined intra- and extracellular bioorthogonal approach, we explored the possibility of enhanced therapeutic efficacy when combining catalysis mediated by PZ-TMA and PZ-COOH. Mitoxantrone is a chemotherapeutic agent that acts by intercalating with DNA and inhibiting the intracellular enzyme topoisomerase II.^34^ We hypothesized that the combined bioorthogonal activation of a chemically quenched derivative of mitoxantrone, both intra- and extracellularly, would ultimately lead to higher intracellular concentrations of mitoxantrone and therefore enhanced cell killing. A quenched derivative of the drug was synthesized by attaching two allyl carbamate moieties (pro-Mitox), thereby efficiently quenching the therapeutic activity of the drug (Fig. 8a).^57^ HeLa cells were seeded on a 96-well plate 24 h prior to the experiment, followed by treatment with PZ-TMA for 24 h. Thereafter, the cells were washed thoroughly with PBS and treated with PZ-COOH and pro-Mitox for an additional 48 h. Finally, the media was removed, and the cell viability was determined using an Alamar blue assay (Fig. 8b). Based on the toxicity assay of the respective nanozymes (Fig. S23), different concentrations of PZ-TMA and PZ-COOH were selected for this step. Additionally, as non-uptaken PZ-TMA was removed before the addition of the prodrug, and pro-Mitox and PZ-COOH were administered simultaneously, a higher concentration of PZ-TMA (800 nM) compared to PZ-COOH (400 nM) was selected for the cell killing studies. Cell killing was observed using the intracellularly active PZ-TMA at high concentrations of pro-Mitox (5 µM). However, more efficient cell killing was observed using the extracellularly active PZ-COOH, presumably due to less protein corona formation compared to PZ-TMA and the direct access to the substrate extracellularly.^58^ Additionally, when half of the concentration of PZ-COOH and PZ-TMA were used in combination, equal cell killing was achieved as with PZ-COOH alone, indicating that combined intra- and extracellular catalysis of pro-Mitox allows for efficient prodrug activation. These findings demonstrate similar cell-killing efficacy using an overall lower nanozyme amount (200 nM PZ-COOH + 400 nM PZ-TMA *vs.* 800 nM PZ-TMA) when both nanozymes were used together. Finally, when both PZs are used at full concentration (PZ-TMA at 800 nM and PZ-COOH at 400 nM), significantly more cell killing was observed compared to the treatment with each PZ alone. However, the combination of both PZs at full concentration resulted in a moderate reduction of HeLa cells without the presence of pro-Mitox, which should be considered for the design of future studies involving intra- and extracellularly acting bioorthogonal PZs.

## Conclusion

In summary, amphiphilic polymer nanoscaffolds can encapsulate transition metal catalysts, creating bioorthogonal PZs that allow access to non-native catalytic reactions. In this study, we engineered the polymer nanoscaffolds to feature a positively or negatively charged PZ surface, impacting the catalytic behavior of the respective PZ within a biological environment. We demonstrated that each respective charged PZ possesses its own differential behavior in the presence of mammalian cells: (1) a positive surface charge of bioorthogonal PZs enhances intracellular uptake compared to a negative surface charge, while (2) a negative surface charge of bioorthogonal PZs likely decreases protein corona formation compared to positively charged PZs and thereby improves catalysis in the presence of serum proteins.

Using a combined intra- and extracellular approach, concurrent catalytic activity enhanced the conversion of chemically quenched imaging agents or therapeutic molecules, thereby increasing the overall accumulation of these molecules within cells, presumably through intracellular activation and simultaneous extracellular activation, followed by substrate diffusion into the cell. We have demonstrated the therapeutic applicability of this approach by performing the concurrent intra- and extracellular activation of a quenched derivative of mitoxantrone. Our results demonstrate enhanced cancer cell killing when intra- and extracellular catalysis are performed simultaneously, indicating that spatial control of catalysis mediated by bioorthogonal PZs can improve the therapeutic outcome of chemotherapy through bioorthogonal prodrug activation.

## Experimental

All chemicals used throughout this study were purchased from either Fisher Scientific or Sigma-Aldrich and used without further purification. All experiments involving HeLa cells were performed in high-glucose DMEM medium supplemented with 10% fetal bovine serum and 1% Antibiotic–Antimycotic (Gibco™). Incubation of HeLa cells was performed at 37 °C and 5% CO_2_, unless otherwise specified.

### Synthesis of pro-Rho and pro-Mitox

Pro-Rho and pro-Mitox were synthesized as previously established in the literature.^57,59^ A brief synthesis protocol and characterization are provided in the SI.

### Polyzyme fabrication and characterization

PZs were prepared by flash nanoprecipitation (FNP) as previously reported.^37^ Briefly, the polymer stock solution of PONI-C_11_-TMA was prepared in MilliQ water at a concentration of 1 mg mL^−1^. A solution of PONI-C_10_-COOH was prepared at a concentration of 1 mg mL^−1^ in MilliQ water with 0.1 mg mL^−1^ NaOH to allow for solubility. The catalyst Pd(dppf)Cl_2_ was dissolved in a 1 : 1 mixture of dimethyl sulfoxide and acetone immediately before FNP at concentrations of 4 mg mL^−1^ for PZ-TMA and 1 mg mL^−1^ for PZ-COOH to accommodate differences in catalyst loading. For PZ fabrication, 0.6 mL of polymer solution was loaded into a syringe, and 0.6 mL of catalyst solution was loaded into another syringe, followed by rapidly impinging the solutions into the FNP mixing chamber and collecting them in a quench bath consisting of 6 mL MilliQ water. Importantly, the quench bath collecting PZ-COOH contained NaBH_4_ (50 µM) to stabilize the resulting negatively charged PZ. Thereafter, the respective PZ solutions were transferred into a centrifuge filter (30 kDa), and the remaining organic solvent was washed out with MilliQ water by centrifugation (three times, 2800 rpm, 21 °C). After centrifugation, the PZ solutions were filtered through a PES syringe filter (0.22 µm), and the weight of the final solution was used to calculate the respective PZ concentration. After obtaining the solutions, PZs were characterized by DLS and *ζ*-potential measurement in MilliQ water at a concentration of 2 µM.

### Transmission electron microscopy (TEM) and energy-dispersive X-ray spectroscopy (EDS)

Samples for TEM observations were prepared on 400 mesh carbon-coated Copper grids (3–5 nm thickness) with additional lacey carbon support (PZ-TMA at 8.5 µM and PZ-COOH at 32 µM, glow discharge for PZ-COOH). Both Bright Field as well as High Angle Annular Dark Field (HAADF) images were recorded using a JEOL JEM-2200FS Energy Filtered S/TEM. EDS was performed with an Oxford XMax-80T EDS detector in scanning transmission mode with a spot size of 1 nm and an acceleration voltage of 200 kV. EDS measurements were performed using a beryllium holder; therefore, only the copper signal from the grid was detected, besides the sample, which does not interfere with the peaks of the elements of interest.

### Catalytic performance and Michaelis–Menten kinetics

The catalytic performance of the respective PZs was determined through the bioorthogonal activation of pro-Rho and monitoring of the fluorescence of the product Rho (ex. 488 nm/em. 521 nm) using a fluorescence microplate reader. First, the respective PZs were diluted in PBS and added to a 96-well plate to obtain a final concentration of 1 µM. Then, the Pro-Rho stock solution was prepared in DMSO at a concentration of 10 mM and diluted with PBS, and added to the plate to obtain a final concentration of 10 µM before monitoring fluorescence. For the Michaelis–Menten kinetics, a 96-well plate was prepared in a similar manner by adding each respective PZ to the plate, obtaining final concentrations of 1 µM and varying the pro-Rho concentration (final concentrations of 10/5/2.5/1.25/0.63/0.31/0.16 µM). The initial velocity was calculated by converting the fluorescence into catalytic yield using a standard curve and plotting it as a function of time. Thereafter, the initial velocity was plotted against the substrate concentration, yielding the Michaelis–Menten constant *K*_M_ and maximum velocity *V*_max_, which was used to determine the catalytic turnover *K*_cat_.

### Catalytic performance in the presence of serum proteins

The catalytic performance of each respective PZ in the presence of serum proteins was determined and compared to the catalytic performance in the absence of serum proteins. Briefly, PZs were fabricated as previously described and incubated in a 96-well plate with FBS (10%) in PBS at a concentration of 2 µM for 10 min. Thereafter, a stock solution of 10 mM pro-Rho in DMSO was dissolved in PBS to reach a final concentration of 20 µM. The solution was then added to the PZ-containing wells of the plate to obtain final concentrations of 1 µM of PZ and 10 µM of pro-Rho. The fluorescence of Rho (ex. 488 nm/em. 521 nm) was measured for 45 min, and the linear slope of the resulting curves was calculated after subtracting the background.

### Intracellular uptake study

The intracellular uptake of PZ-TMA and PZ-COOH in HeLa cells was determined over time. Briefly, HeLa cells were plated in a 24-well plate (20k cells per well) 24 h prior to the experiment. Thereafter, the cells were incubated with the respective PZs at different concentrations (300, 500, or 700 nM) in medium for different durations. After incubation, the cells were washed three times with PBS before the addition of lysis buffer. After cell lysis, the cells were transferred into 15 mL Falcon tubes and digested with aqua regia for 24 h before quantifying the amount of Pd^106^ using ICP-MS. Each experimental condition was performed in triplicate.

### Intracellular catalytic activity

The intracellular catalytic conversion of pro-Rho was quantified in HeLa cells using two different experimental protocols. Initially, HeLa cells were seeded on a black 96-well plate (10k cells per well) 24 h prior to each experiment. Then, HeLa cells were incubated for 3 h with the respective PZs at different concentrations (300/500/700 nM) before washing the cells three times with PBS, adding pro-Rho (40 µM), and monitoring the fluorescence of Rho (ex. 488 nm/em. 521 nm) for 2 h. Thereafter, the experiment was performed in reverse order by incubating HeLa cells with pro-Rho (100 µM) for 3 h, followed by washing the cells with PBS three times and addition of the respective PZ at varying concentrations (300/500/700 nM). The fluorescence of Rho was monitored for 2 h.

### Determination of intra-*versus* extracellular catalytic activity

The intracellular fluorescence of Rho was determined by comparing either intracellular activation mediated by PZ-TMA, extracellular activation mediated by PZ-COOH, or a combination of both. Briefly, HeLa cells were seeded in a black 96-well plate (10k cells per well) 24 h prior to the experiment. The cells were then incubated with PZ-TMA for 3 h before washing them three times with PBS, followed by the addition of PZ-COOH and pro-Rho (100 µM). The fluorescence of Rho was measured after an additional 3 h before removing the medium, as well as after removing the medium and adding fresh PBS.

### Combination study on HeLa cell viability

The therapeutic potential of the concurrent intra- and extracellular bioorthogonal prodrug activation was demonstrated on HeLa cells. Briefly, HeLa cells were seeded in a 96-well plate (3k cells per well) 24 h prior to the experiment. The cells were incubated with PZ-TMA (800 nM) for 24 h, followed by washing them three times with PBS. Thereafter, PZ-COOH (400 nM) and pro-Mitox at different concentrations (0, 3, and 5 µM) were added and incubated for an additional 48 h. The medium was then removed, and cell viability was determined using an AlamarBlue™ assay (Invitrogen).

## Author contributions

Conceptualization, C.-M. H., Y. A. C., V. M. R.; investigation, C.-M. H., M. S., Y. A. C.; synthesis, C.-M. H., J. Y., J. T., M. A.; characterization, C.-M. H., N. N., A. R.; writing, C.-M. H., M. S.; review and editing, all authors; funding acquisition and resources, V. M. R.

## Conflicts of interest

There are no conflicts to declare.

## Supplementary Material

SC-OLF-D5SC07223A-s001

## Data Availability

The data supporting this article have been included as part of the supplementary information (SI). Supplementary information is available. See DOI: https://doi.org/10.1039/d5sc07223a.
