# Supplementary material for: Controlled intra- and extracellular localization of bioorthogonal polymeric nanozymes
Source: Chem Sci. 2026 Jan 2;17(8):4050–60. doi: 10.1039/d5sc07223a (PMC12776530; doi:10.1039/d5sc07223a)
Supplement: SC-017-D5SC07223A-s001 [file SC-017-D5SC07223A-s001.pdf]

Supplementary Information (SI) for Chemical Science.
This journal is © The Royal Society of Chemistry 2025

Supporting Information

Controlled Intra- and Extracellular Localization of Bioorthogonal Polymeric Nanozymes

Cristina-Maria Hirschbiegel, Mathangi Shrikanth, Yagiz Anil Cicek, Nourina Nasim, Joe Truong, Junwhee Yang, Alexander Ribbe, Maged Abdelaziz, and Vincent M. Rotello

Contents

1. Synthesis and characterization of pro-Rho 2
2. Synthesis and characterization of pro-Mitox 2
3. Synthesis and characterization of PONI-C_{11}-TMA 3
4. Synthesis and characterization of PONI-C_{10}-COOH 9
5. Energy-dispersive X-ray spectroscopy (EDS) of polyzymes 13
6. Inductively coupled plasma mass spectrometry (ICP-MS) 15
7. Michaelis-Menten plots 16
8. HeLa cell viability of polyzymes 17

1. Synthesis and characterization of pro-Rho

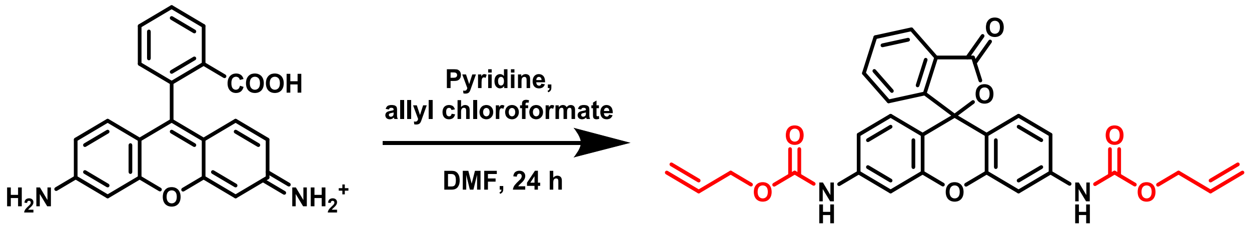

Figure S1. Synthetic scheme of pro-Rho synthesis.

Pro-Rho was synthesized according to a modified version of a reported protocol.[1] Briefly, rhodamine 110 (50 mg, 0.14 mmol) and pyridine (40 µL, 0.46 mmol) were dissolved in dry DMF (3 mL) in a round-bottom flask before purging the solution with nitrogen. Thereafter, the flask was cooled in an ice bath, and allyl chloroformate (35 µL, 0.3 mmol) was added dropwise under stirring. The solution was then left to warm to room temperature and stirred overnight, and the completion of the reaction was monitored by thin-layer chromatography. After completion, ethyl acetate (15 mL) was added to the solution, which was then washed three times with 5% hydrochloric acid (5 mL) and subsequently three times with a saturated sodium bicarbonate solution (5 mL). The organic layer was collected and dried using sodium sulfate before concentrating it under reduced pressure. Finally, the product was purified by column chromatography using a gradient column (10:1 to 2:1 hexane/ethyl acetate), yielding a white solid (21 mg, 28% yield).

^{1}H-NMR (400 MHz, CDCl3): δ (ppm) = 8.04 (d, 1H), 7.66 (dtd, 2H), 7.51 (d, 2H), 7.16 (dd, 1H), 6.98 (dd, 2H), 6.87 (s, 2H), 6.73 (d, 2H), 5.99 (m, 2H), 5.42 (q, 1H), 5.37 (q, 1H), 5.30 (dq, 2H), 4.71 (dt, 4H).

2. Synthesis and characterization of pro-Mitox

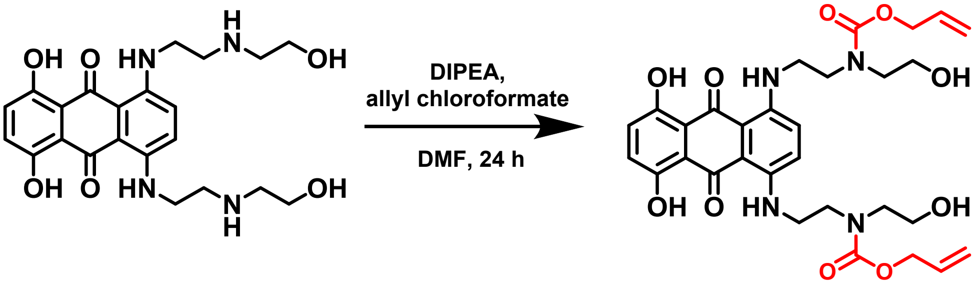

Figure S2. Synthetic scheme of pro-Mitox synthesis.

Pro-Mitox was synthesized according to a previously reported protocol.[2] Briefly, mitoxantrone (50 mg, 0.11 mmol) was dissolved in dry DMF (3 mL) in a round-bottom flask before purging the solution with nitrogen and cooling it in an ice bath, followed by the addition of diisopropylethylamine (3 eq., 60 µL) and dropwise addition of allyl chloroformate (2 eq, 25 µL). The solution was then left to warm to room temperature and stirred overnight, and the

completion of the reaction was monitored by thin-layer chromatography. After completion, the solution was diluted with water (10 mL), and the product was extracted with ethyl acetate (10 mL) three times, before drying the organic layer with sodium sulfate and concentrating it under reduced pressure. Pro-Mitox was purified by column chromatography using a gradient column (10:1 to 1:1 hexane/ethyl acetate), yielding a blue solid (22 mg, 32% yield).

^{1}H-NMR (400 MHz, CDCl3): δ (ppm) = 13.29 (s, 2H), 10.23 (s, 2H), 7.09 (s, 2H), 7.02 (s, 2H), 6.00 (d, 2H), 5.37 (m, 2H), 5.27 (d, 2H), 4.67 (t, 4H), 3.83 (t, 4H), 3.65-3.56 (m, 12H).

3. Synthesis and characterization of PONI-C_{11}-TMA

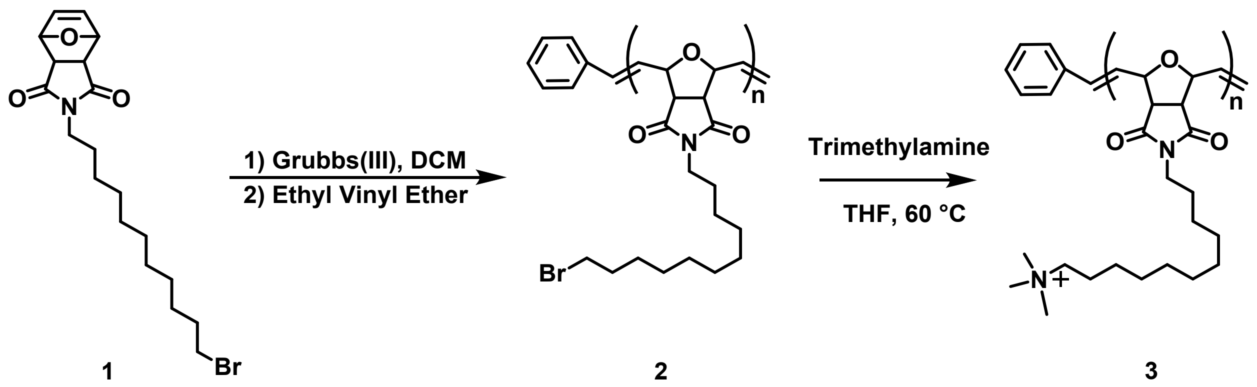

Figure S3. Synthetic scheme of PONI-C_{11}-TMA (3) synthesis.

Synthesis of 1: The oxanorbornene derivative **1** was synthesized by Diels-Alder cycloaddition using maleimide and furan, followed by a substitution reaction with undecane bromide as described in the literature.[3] Briefly, Furan (4.5 mL, 61.7 mmol, 1.5 equiv) and maleimide (4.0 g, 41.1 mmol, 1.0 equiv) were dissolved in 5 mL of diethyl ether in a pressure tube. The solid product was cooled at room temperature and collected by filtration, washed thoroughly with diethyl ether. In a 250 mL round-bottom flask equipped with a stir bar, a portion of previous crude product (2.50 g, 15.1 mmol, 1.0 equiv) was dissolved in 39.9 mL DMF in a 250 mL. Potassium carbonate (8.37 g, 60.6 mmol, 4.0 equiv) was mixed with the crude and heated at 50 °C for 5 min. A mixture of potassium iodide (0.45 g, 2.99 mmol, 0.2 equiv) and 11-bromoundecanol (3.99 g, 15.9 mmol, 1.05 equiv) were added, and the reaction was stirred at 50 °C overnight. After cooling the mixture to room temperature, it was diluted with 100 mL ethyl acetate, washed with water and brine, dried over sodium sulfate, and concentrated under reduced pressure. In a round-bottom flask containing 41.7 mL of DCM, a portion of the second crude product (1.10 g, 3.28 mmol, 1.0 equiv) was cooled in an ice bath. A mixture of tetrabromomethane (1.30 g, 3.93 mmol, 1.2 equiv) and triphenylphosphine (1.03 g, 3.93 mmol, 1.2 equiv) were added. The mixture was stirred at 0 °C for 3 h, concentrated by rotary evaporation, diluted with 200 mL ethyl ether, and placed in the freezer for 2 h to precipitate triphenylphosphine oxide. After filtration, the filtrate was concentrated and purified by column chromatography to afford compound **1** as a white solid.

^{1}H-NMR (400 MHz, $CDCl_3$): δ (ppm) = 6.53 (s, 2H), 5.29 (s, 2H), 3.44 (t, 2H), 3.41 (t, 2H), 2.85 (s, 2H), 1.87 (q, 2H), 1.57 (q, 2H), 1.41 (q, 2H), 1.28 (m, 12H).

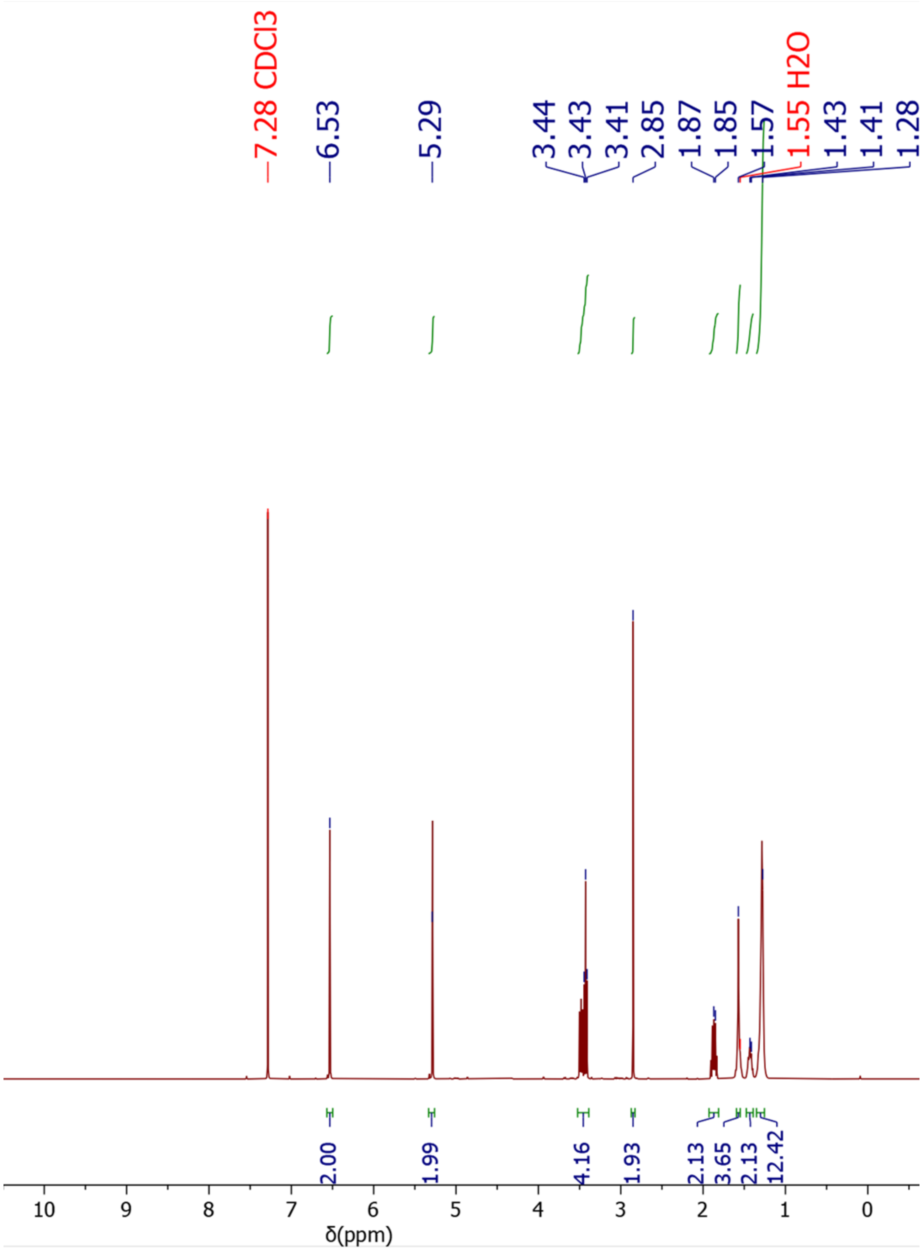

Figure S4. ^{1}H-NMR spectrum of compound **1** in $CDCl_3$ (400 MHz).

Synthesis of 2: For the polymer synthesis, monomer (compound **1**) (250 mg, 0.625 mmol, 1.0 eq) was dissolved in dry 3 mL DCM inside a 10 mL pear-shaped, air-free flask fitted with a magnetic stir bar. In a second flask, Grubbs' third-generation catalyst (7.9 mg, 0.0089 mmol,

0.02 eq) was dissolved in DCM. Both flasks were sealed with septa, connected to a Schlenk nitrogen/vacuum line, and underwent three freeze-pump-thaw cycles. After degassing the flasks, they were allowed to warm to room temperature, and the catalyst solution was transferred *via* syringe to the reaction mixture. After 30 minutes, 200 μL ethyl vinyl ether was added, and the solution was allowed to stir for 15 minutes to terminate the polymerization process. The crude product was purified using column chromatography with a scavenger (DMT-functionalized silica) to remove the ruthenium catalyst, yielding the polymer (compound **2**). The molecular weight of compound **2** was determined (28,800 Da, PDI = 1.06) using THF-GPC and a polystyrene calibration.

^{1}H-NMR (400 MHz, $CDCl_3$): δ (ppm) = 6.11 (br, 1H), 5.81 (br, 1H), 5.06 (br, 1H), 4.48 (br, 1H), 3.58 (br, 2H), 3.32 (br, 2H), 1.87 (q, 2H), 1.58 (br, 2H), 1.46 (br, 2H), 1.30 (br, 12H).

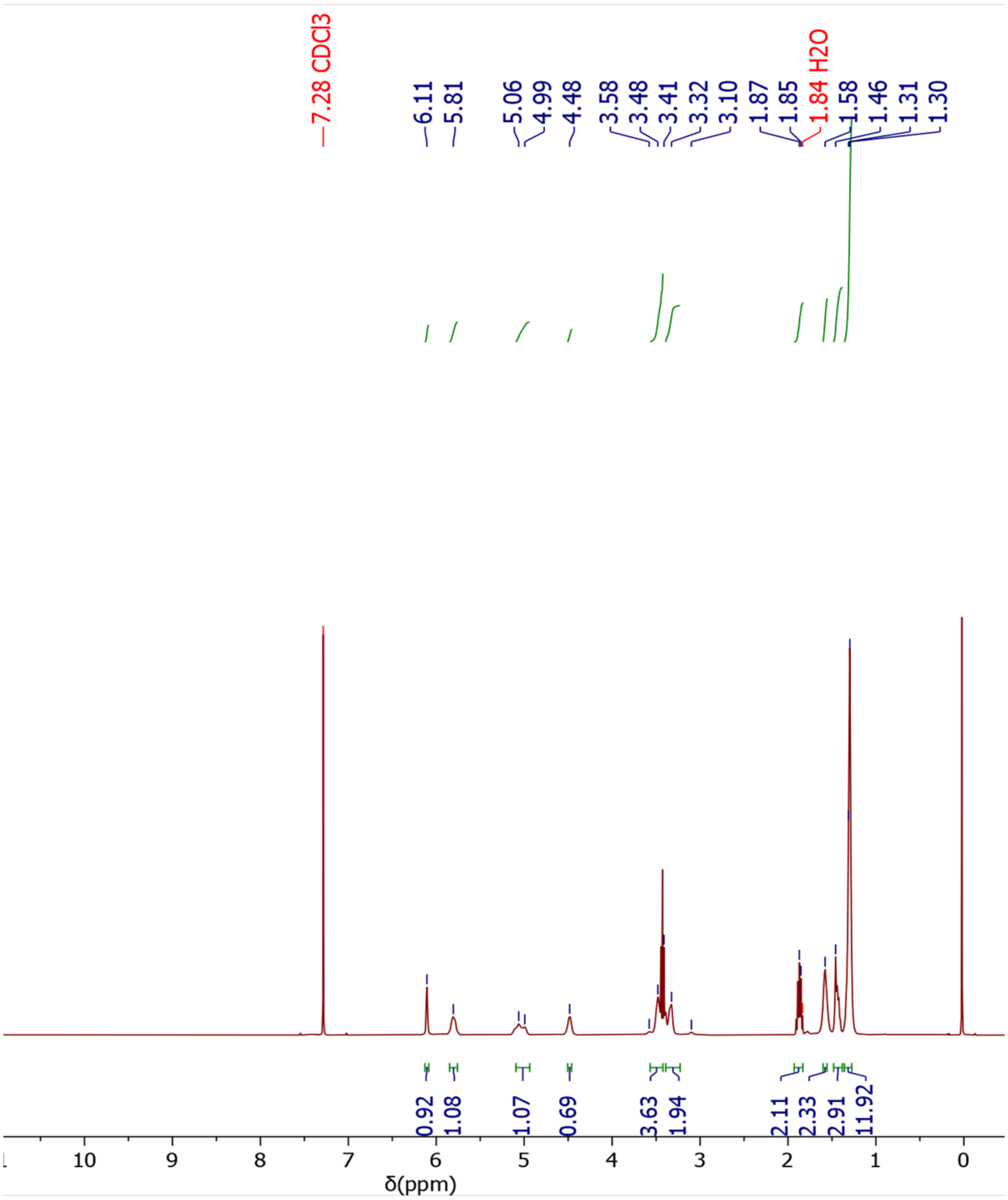

Figure S5. ^{1}H-NMR spectrum of compound **2** in $CDCl_3$ (400 MHz).

Synthesis of 3: Quaternary ammonium poly(oxanorborneneimide) was synthesized by adding an excess amount of trimethylamine, 5 mL of 2 M trimethylamine in tetrahydrofuran to compound **2** (40 mg) to 20 ml vials that had a stir bar. The mixtures were purged with nitrogen, sealed, and heated at 80 °C with stirring for 1 hour, during which the polymers precipitated. Afterward, half of the THF was removed by evaporation and replaced with methanol, allowing the polymers to redissolve. Reactions were continued overnight at 60 °C. The solvents were then fully evaporated, and the crude polymers were washed twice with hexane before being dissolved in a

minimal amount of water. Samples were placed into dialysis membranes (10,000 MWCO) and dialyzed against water for three days with periodic solvent changes. Finally, the solutions were passed through PES syringe filters and lyophilized, yielding PONI-C_{11}-TMA (compound **3**). ^{1}H-NMR confirmed successful conversion to quaternary ammonium salts. Gel Permeation Chromatography (GPC) of compound **2** in THF was performed at 40 °C with a flow rate of 1.0 mL min^{-1} on an Agilent 1260 Infinity system, equipped with a G1362A refractive index detector, a G1310B isocratic pump, three PLgel 5 µm mixed-C columns (7.5 × 300 mm), and a 5 µm guard column (7.5 × 50 mm). The system was calibrated against a polystyrene (PS) standard.

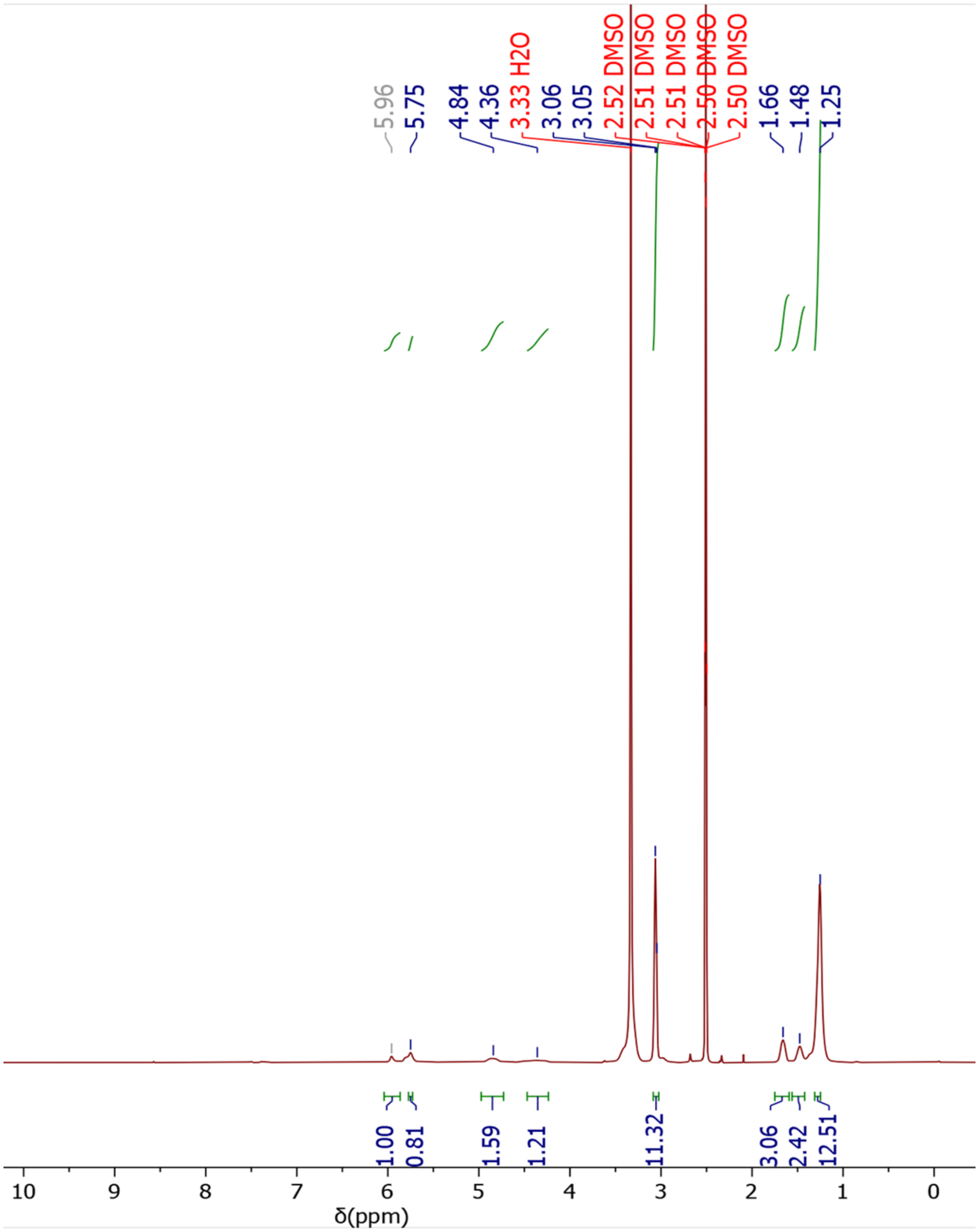

Figure S6. ^{1}H-NMR spectrum of compound **3** in d^6-DMSO (400 MHz).

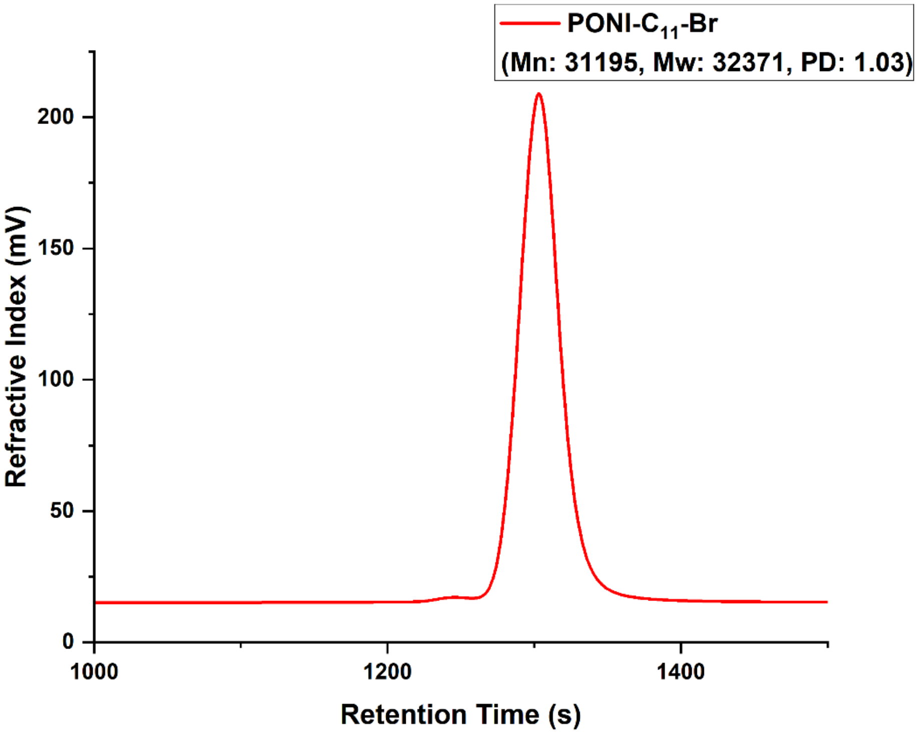

Figure S7. GPC of PONI-C_{11}-Br (compound **2**).

4. Synthesis and characterization of PONI-C_{10}-COOH

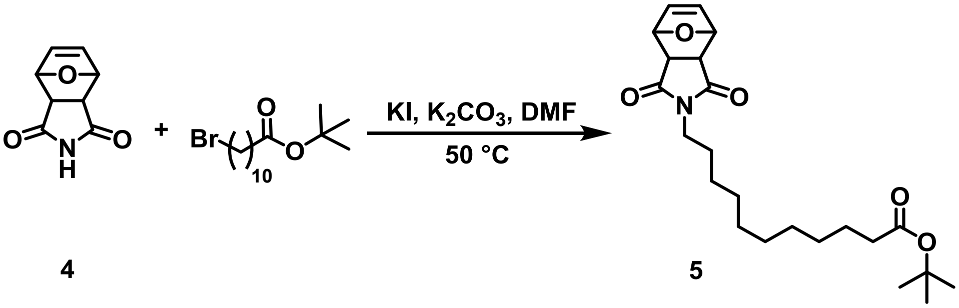

Figure S8. Synthetic scheme of protected monomer **5** synthesis.

Synthesis of 5: To a round-bottom flask, compound **4** (207 mg, 1.12 mmol) and t-butyl 11-bromoundecanoate (387 mg, 1.20 mmol) were dissolved in N,N-dimethylformamide. Then, potassium carbonate (560 mg, 4.05 mmol) and potassium iodide (1.57 mg, 0.024 mmol) were added to the reaction mixture after 15 min of stirring. The reaction was left to stir overnight at 50 °C. The crude product was washed with deionized water (3 x 20 mL) and brine (1 x 20 mL). The organic layer was dried with magnesium sulfate and concentrated *in vacuo*. Then, the product

was purified using column chromatography with 1:1 (hexane/ethyl acetate), yielding compound **5** as a white solid (67%).

^{1}H-NMR (400 MHz, $CDCl_3$): δ (ppm) = 6.53 (s, 2H), 5.28 (s, 2H), 3.47 (t, 2H, J = 8 Hz), 2.84 (s, 2H), 2.21 (t, 2H, J = 8 Hz), 1.61-1.54 (m, 4H), 1.46 (s, 9H), 1.28 (s, 12H).

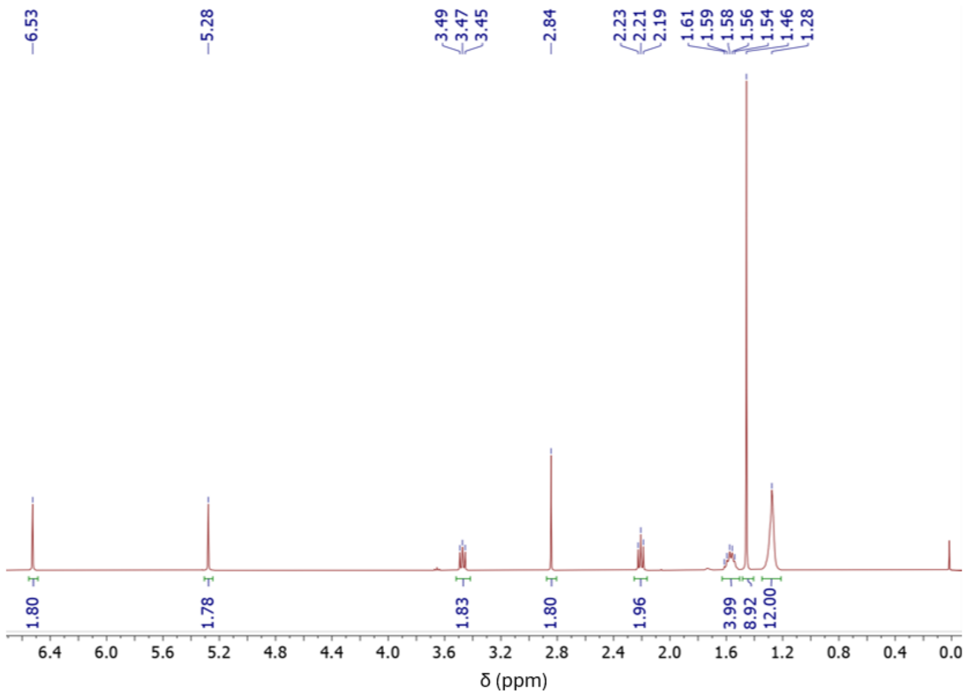

Figure S9. ^{1}H-NMR spectrum of compound **5** in $CDCl_3$.

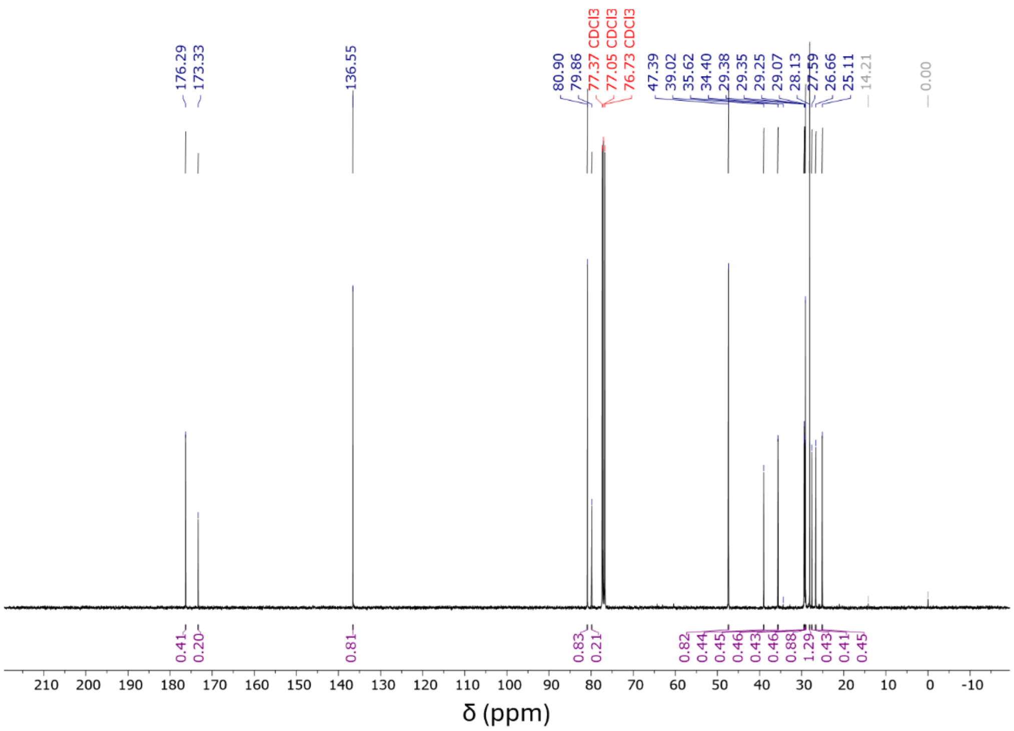

Figure S10. ^{13}C-NMR spectrum of compound **5** in $CDCl_3$ (400 MHz).

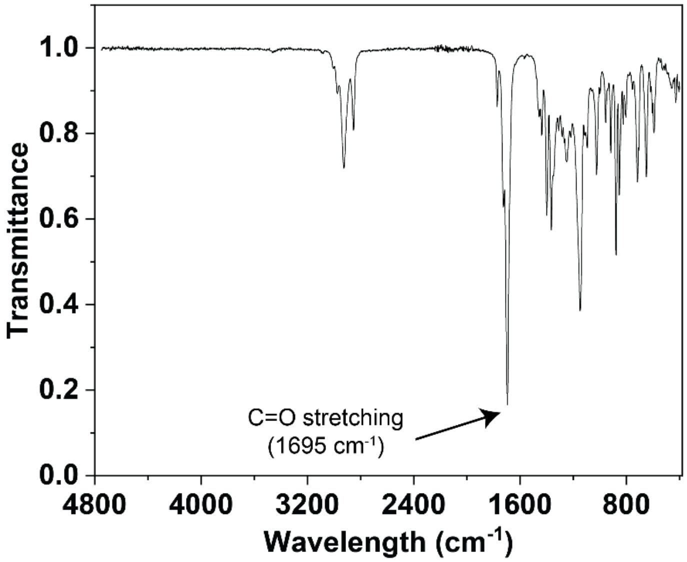

Figure S11. Transmission spectrum of compound **5** (solid).

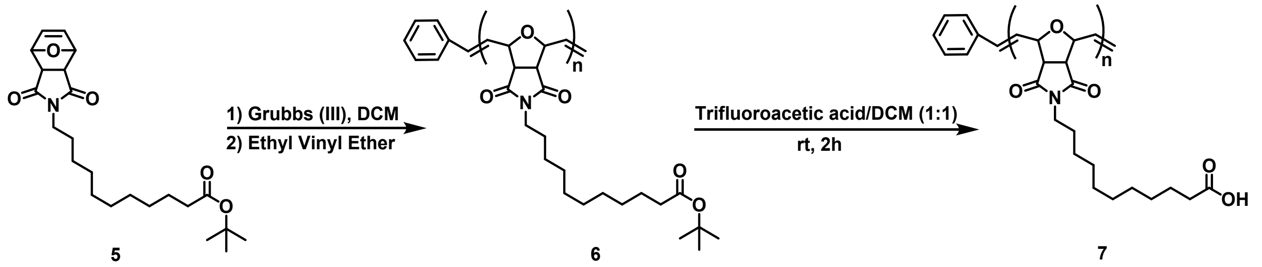

Figure S12. Synthetic scheme of PONI-C_{10}-COOH synthesis (compound **7**).

Synthesis of 7: To a round-bottom flask, compound **5** (100 mg, 0.247 mmol) was dissolved in dichloromethane. Then, Grubbs' third-generation catalyst (2.73 mg, 0.00309 mmol) was added to the reaction mixture with stirring. After 30 min, the reaction was cleaved with the addition of 200 µL ethyl vinyl ether. The polymer (compound **6**) was purified using aluminum oxide column chromatography, then concentrated *in vacuo*. The polymer was then deprotected using 8 mL of trifluoroacetic acid in DCM (1:1) at 70 °C for 2 h in a sealed vial. Compound **7** was obtained as a white polymer after concentration in vacuo overnight (95% yield). The molecular weight of compound **7** was determined by GPC using polystyrene as a standard (38 kDa), following the same protocol as described for compound **2**.

^{1}H-NMR (400 MHz, D_2O): δ (ppm) = 5.90 (br, 2H), 3.39 (br, 2H), 2.08 (3H), 1.21 (br, 17H).

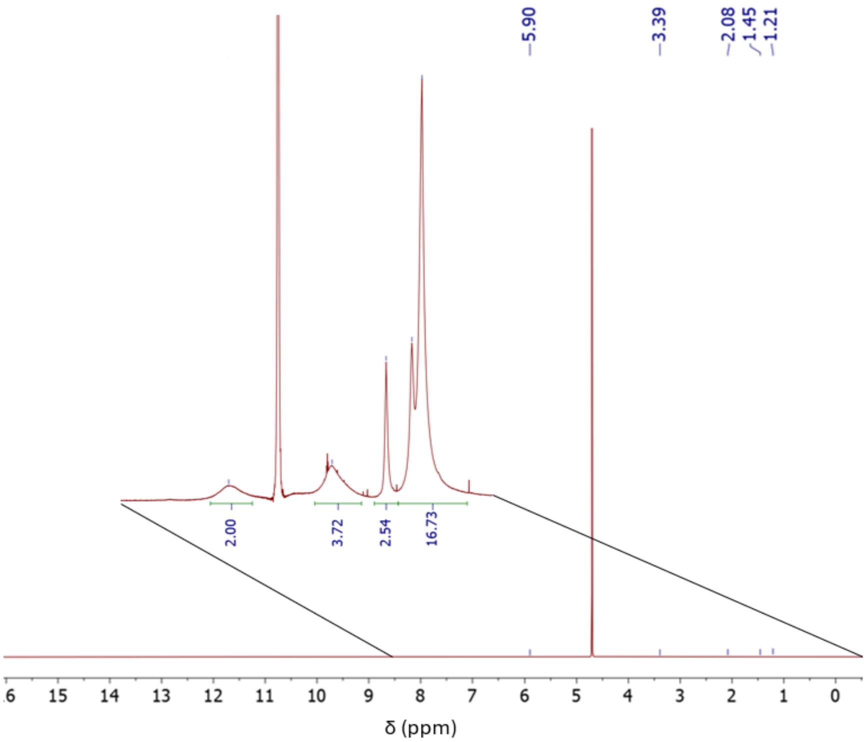

Figure S13. ^{1}H-NMR spectrum of compound **7** in D_2O (400 MHz).

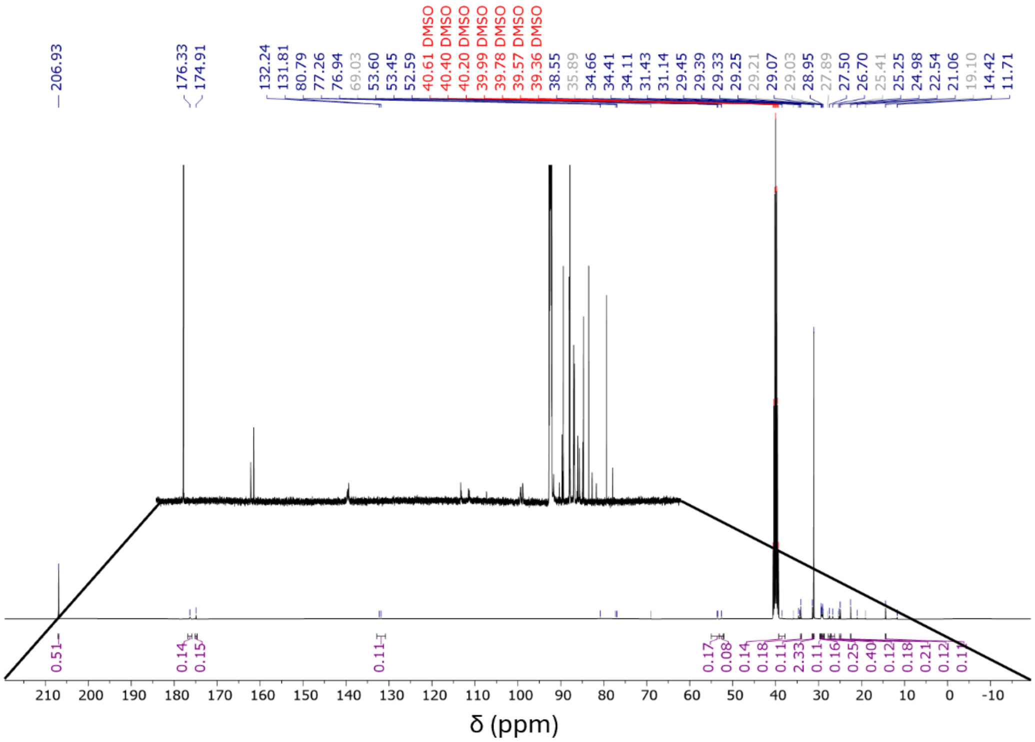

Figure S14. ^{13}C-NMR spectrum of compound **7** in D_6-DMSO (400 MHz).

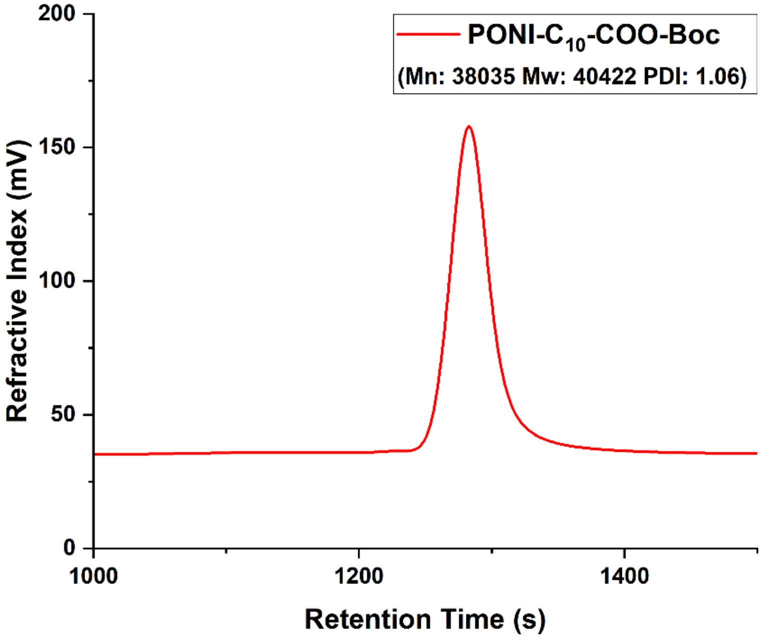

Figure S15. GPC of PONI-C_{10}-COO-Boc (compound **6**).

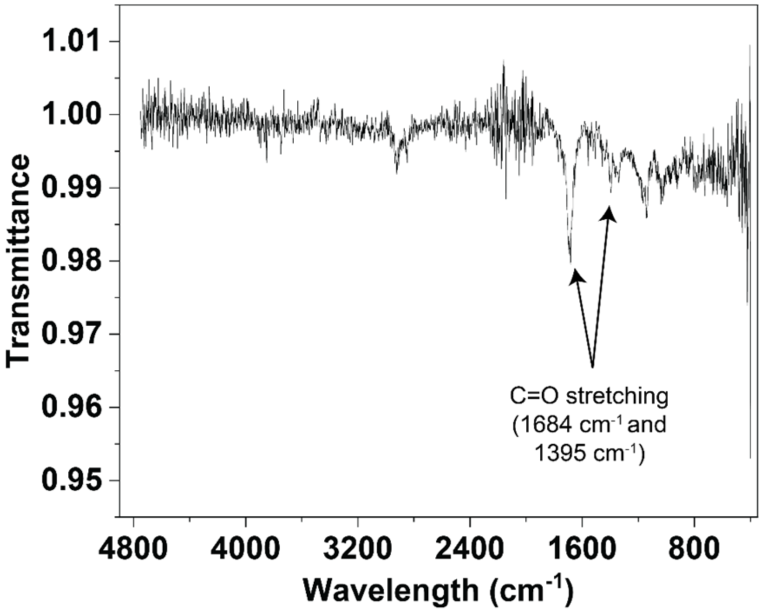

Figure S16. Transmission spectrum of compound **7** (solid).

5. Energy-dispersive X-ray spectroscopy (EDS) of polyzymes

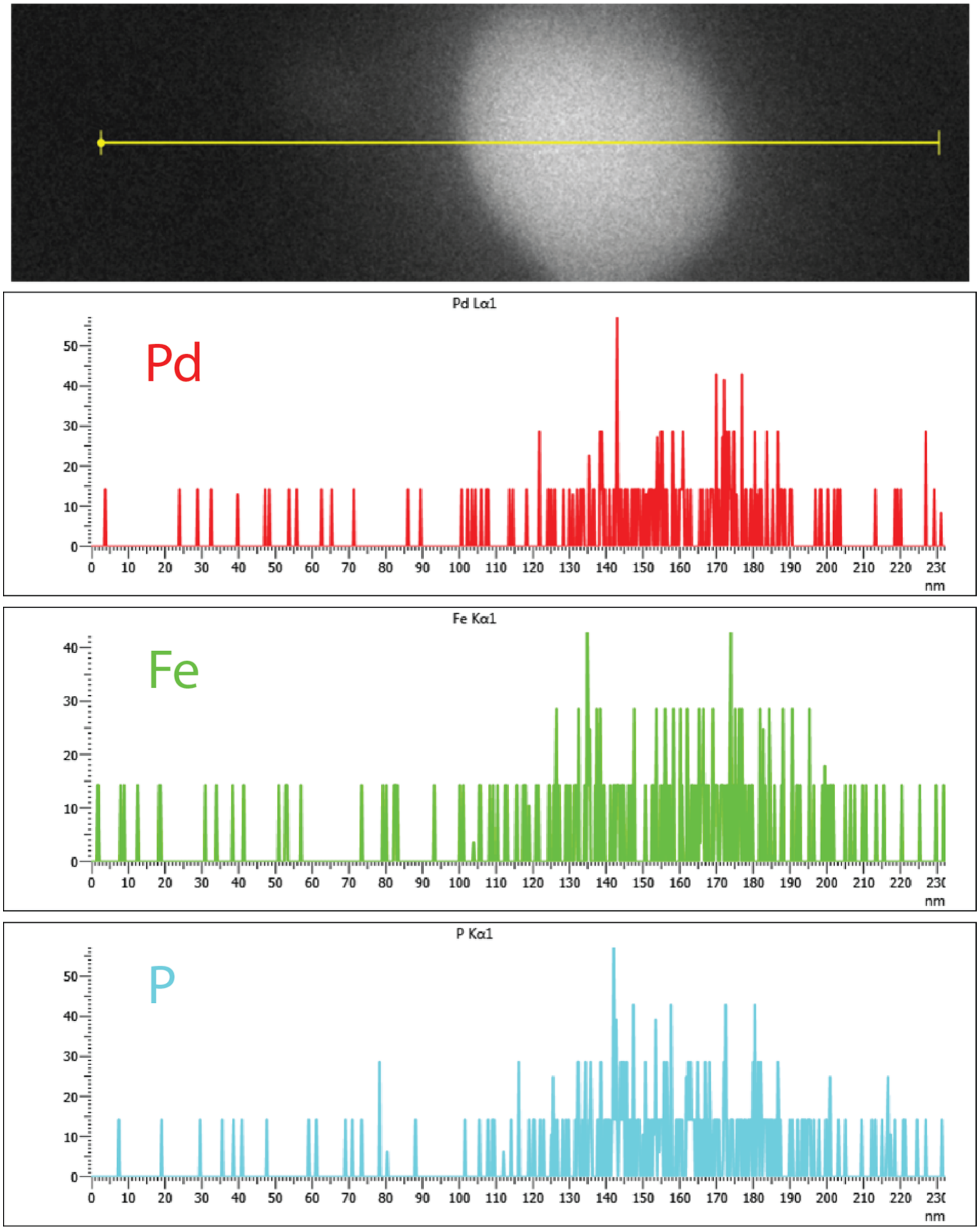

Figure S17. EDS line scans of Pd, Fe, and P within PZ-TMA.

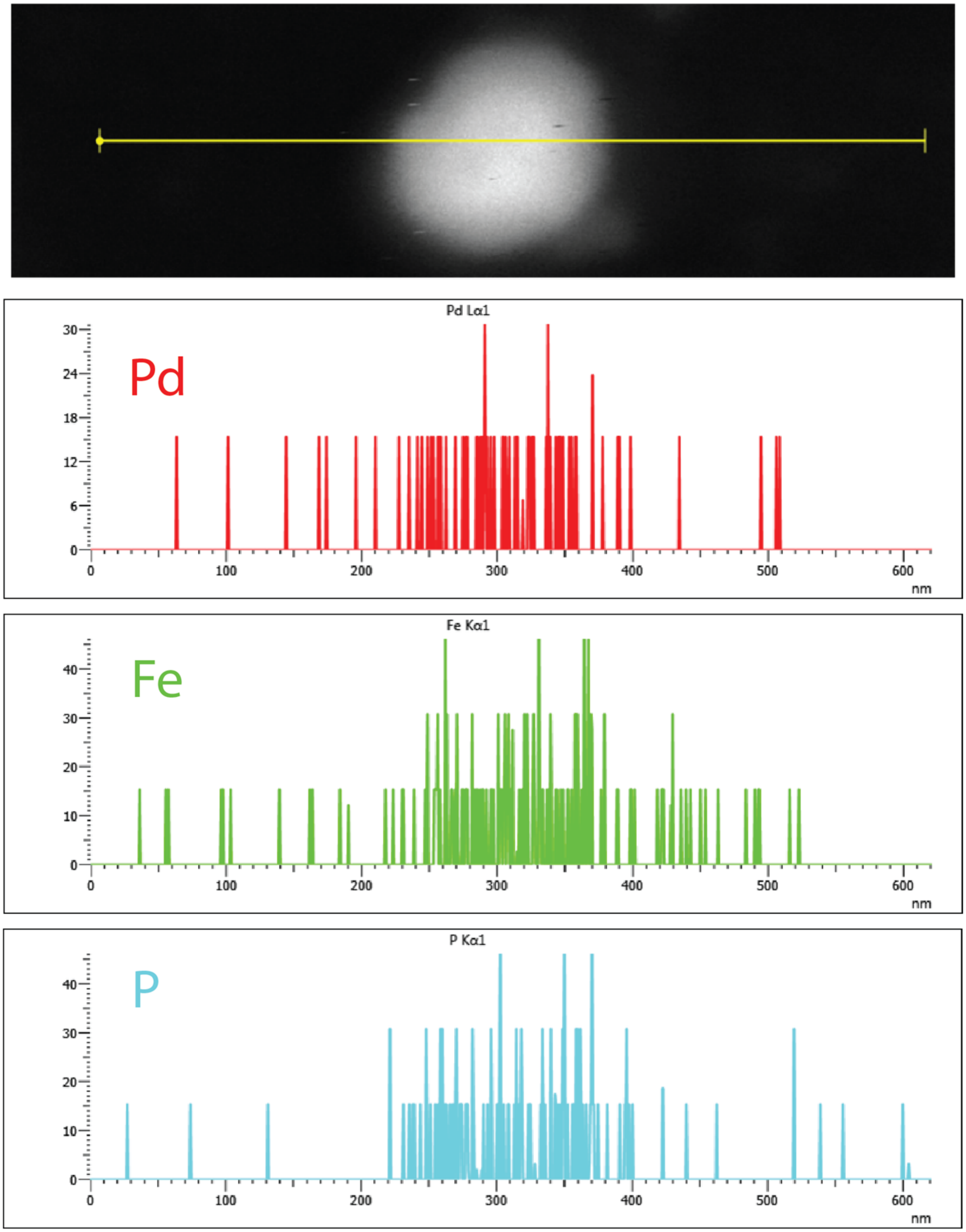

Figure S18. EDS line scans of Pd, Fe, and P within PZ-COOH.

6. Inductively coupled plasma mass spectrometry (ICP-MS)

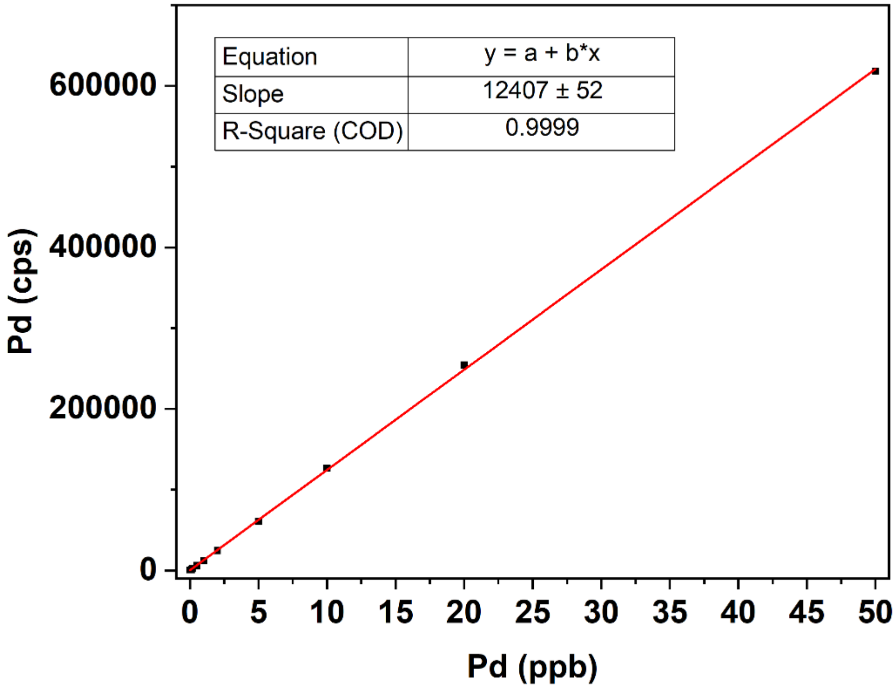

Figure S19. ICP standard curve for Pd^{106}.

Catalyst encapsulation and nanozyme stability were determined using ICP-MS by creating fresh nanozymes (day 0), storing them at room temperature in the dark for 5 days, and subsequently filtering the nanozyme solution through a 0.22 μm PES syringe filter to remove any precipitated catalyst (day 5). PZ-COOH was stored in MilliQ water containing 0.1 mg/mL NaOH to avoid precipitation of the polymer over time. The respective samples were digested using aqua regia and diluted with MilliQ water before measuring the Pd content of each respective sample.

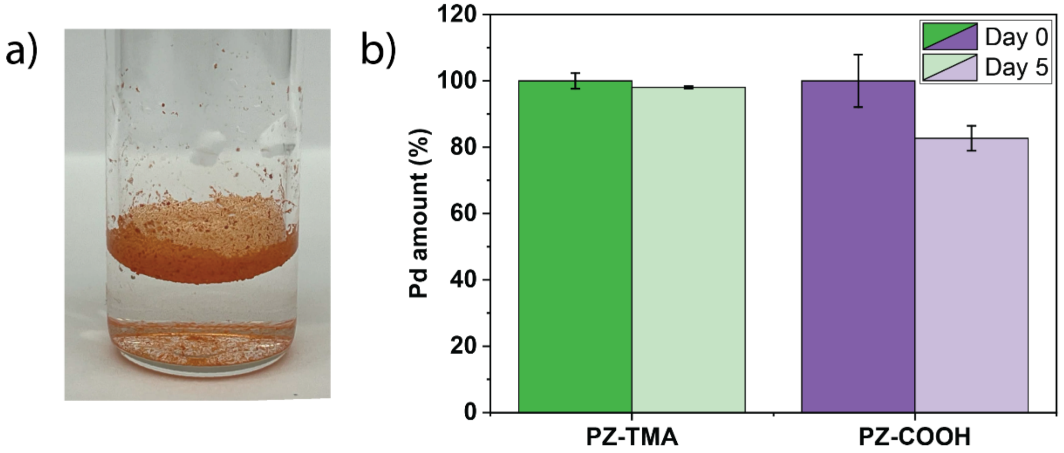

Figure S20. a) Pd catalyst (red) is insoluble in water; b) encapsulated Pd catalyst inside PZ-TMA and PZ-COOH after storage for 5 days in MilliQ water (PZ-TMA) or MilliQ water with 0.1 mg/mL NaOH (PZ-COOH) at room temperature and filtering, quantified by ICP-MS.

7. Michaelis-Menten plots and catalytic activity

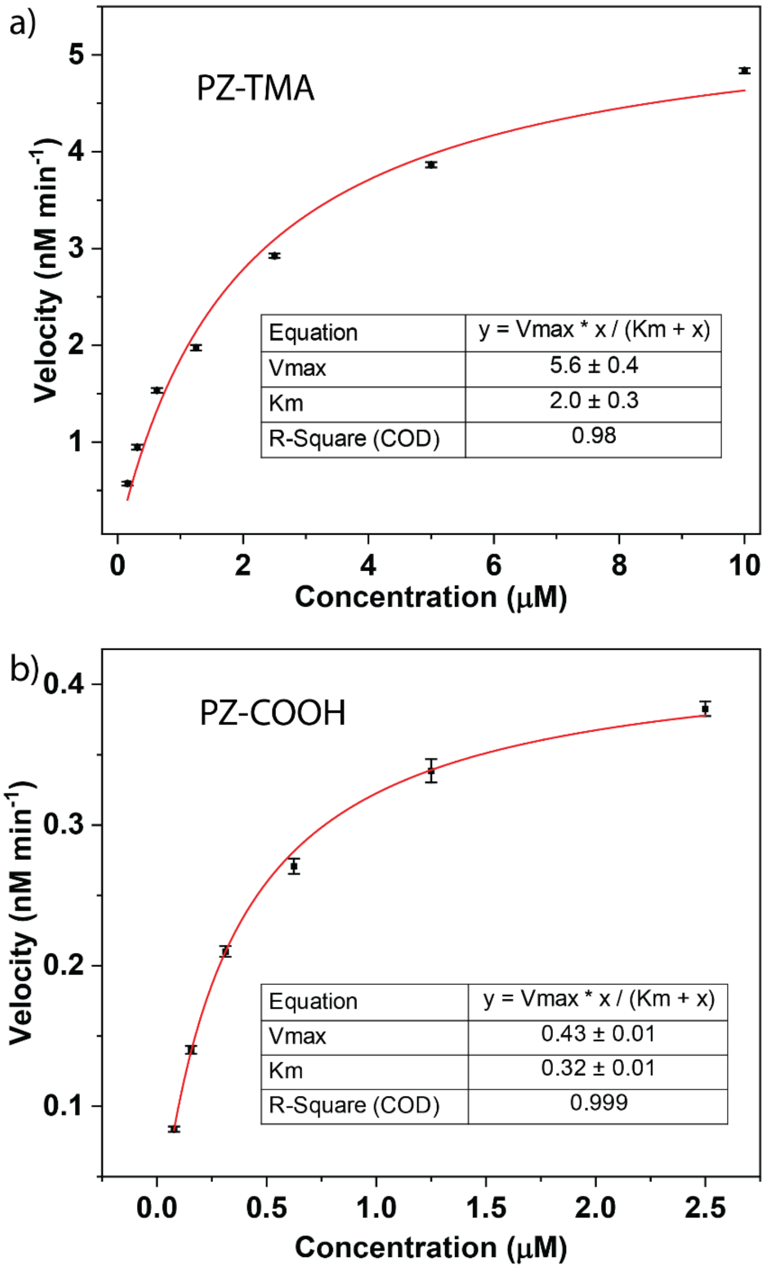

Figure S21. Michaelis-Menten plots of a) PZ-TMA and b) PZ-COOH with pro-Rho.

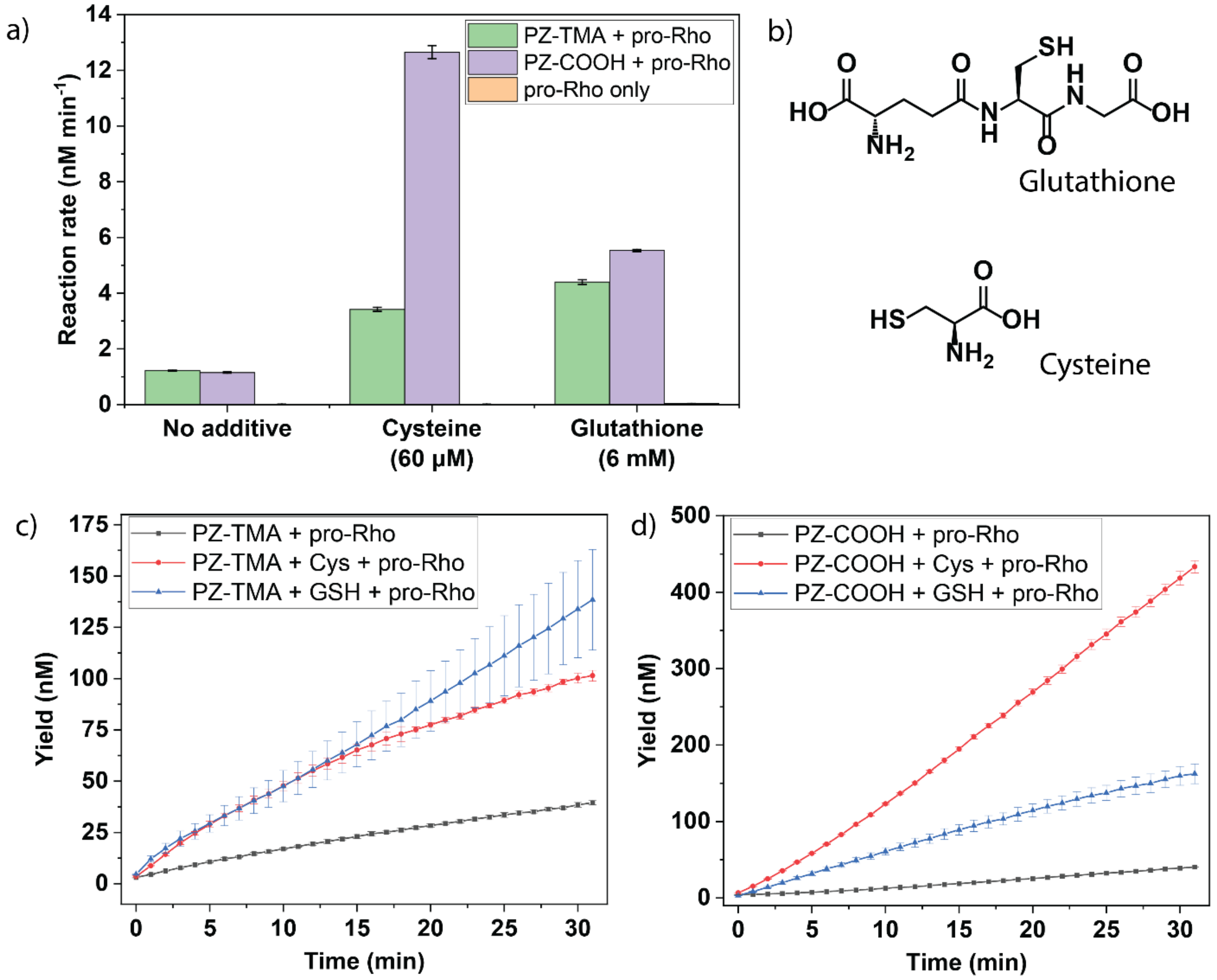

Figure S22. a) Reaction rate of PZ-TMA and PZ-COOH in the absence of additives or presence of either glutathione or cysteine; b) structures of glutathione (GSH) and cysteine (Cys). Reaction kinetics in the presence or absence of glutathione and cysteine of either c) PZ-TMA or d) PZ-COOH.

8. HeLa cell viability of polyzymes

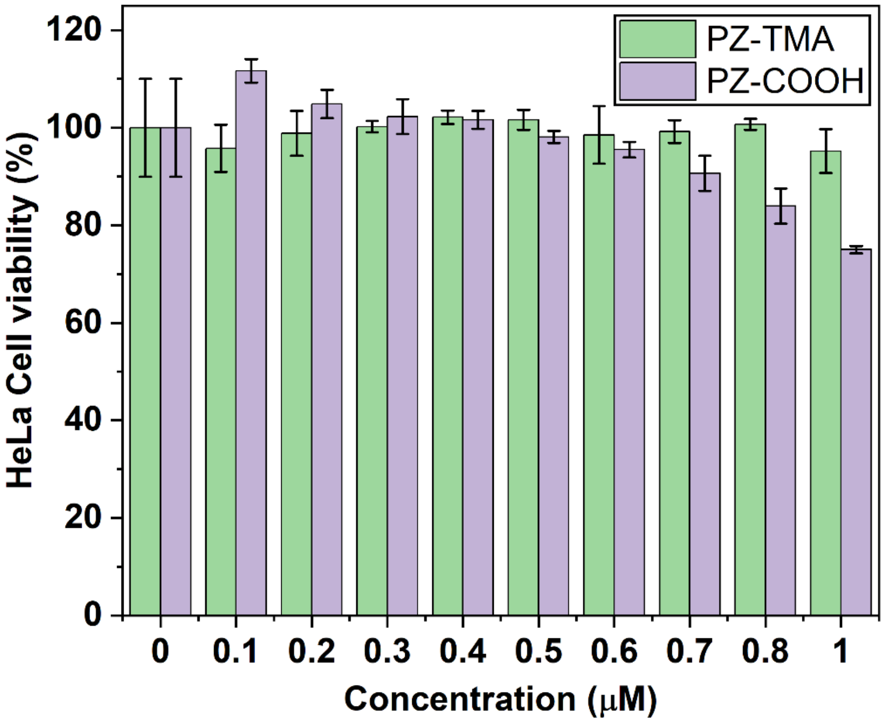

Figure S23. Cell viability of HeLa cells after incubation with PZ-TMA or PZ-COOH for 24 h (10k cells per well).

1. C. Streu and E. Meggers, *Angew. Chem. Int. Ed.*, 2006, **45** 5645-5648.
2. X. Zhang, R. F. Landis, P. Keshri, R. Cao-Milan, D. C. Luther, S. Gopalakrishnan, Y. Liu, R. Huang, G. Li, M. Malassine, I. Uddin, B. Rondon and V. M. Rotello, *Adv. Healthcare Mater.*, 2020, **10**, 2001627.
3. A. Gupta, R. F. Landis, C.-H. Li, M. Schnurr, R. Das, Y.-W. Lee, M. Yazdani, Y. Liu, A. Kozlova and V. M. Rotello, *JACS*, 2018, **140**, 12137-12143.
